# The *Barley stripe mosaic virus* γb protein promotes chloroplast-targeted replication by enhancing unwinding of RNA duplexes

**DOI:** 10.1371/journal.ppat.1006319

**Published:** 2017-04-07

**Authors:** Kun Zhang, Yongliang Zhang, Meng Yang, Songyu Liu, Zhenggang Li, Xianbing Wang, Chenggui Han, Jialin Yu, Dawei Li

**Affiliations:** State Key Laboratory of Agro-Biotechnology and Ministry of Agriculture Key Laboratory of Soil Microbiology, College of Biological Sciences, China Agricultural University, Beijing, P. R. China; University of California, Davis Genome Center, UNITED STATES

## Abstract

RNA viruses encode various RNA binding proteins that function in many steps of viral infection cycles. These proteins function as RNA helicases, methyltransferases, RNA-dependent RNA polymerases, RNA silencing suppressors, RNA chaperones, movement proteins, and so on. Although many of the proteins bind the viral RNA genome during different stages of infection, our knowledge about the coordination of their functions is limited. In this study, we describe a novel role for the *Barley stripe mosaic virus* (BSMV) γb as an enhancer of αa RNA helicase activity, and we show that the γb protein is recruited by the αa viral replication protein to chloroplast membrane sites of BSMV replication. Mutagenesis or deletion of γb from BSMV resulted in reduced positive strand (+) RNAα accumulation, but γb mutations abolishing viral suppressor of RNA silencing (VSR) activity did not completely eliminate genomic RNA replication. In addition, *cis*- or *trans*-expression of the *Tomato bushy stunt virus* p19 VSR protein failed to complement the γb replication functions, indicating that the direct involvement of γb in BSMV RNA replication is independent of VSR functions. These data support a model whereby two BSMV-encoded RNA-binding proteins act coordinately to regulate viral genome replication and provide new insights into strategies whereby double-stranded viral RNA unwinding is regulated, as well as formation of viral replication complexes.

## Author summary

In order to maximize reproduction, RNA viruses often encode multifunctional proteins to perform different activities during replication. We report that the *Barley stripe mosaic virus* γb VSR protein is recruited to viral replication sites through interactions with the αa replicase subunit protein where it enhances αa helicase activity to increase the levels of BSMV replication. Our discovery of the γb RNA helicase enhancement activity provides a novel function associated with a viral suppressor of RNA silencing that enhances understanding of multifunctional strategies whereby virus-encoded proteins contribute to genome replication and counter host defense mechanisms.

## Introduction

Positive sense ssRNA viruses consist of one or more highly structured genomic (g) RNAs. Complex secondary motifs within viral gRNAs are required for efficient viral protein translation [1, 2], RNA synthesis [3, 4], cell-to-cell movement [5] and encapsidation [6, 7]. Current virology studies have shown that RNA viruses use several strategies to regulate secondary RNA structures during replication, including RNA helicase and RNA chaperone functions. For example, conserved RNA helicase domains are found within many plant and animal viral proteins (S1 Fig). These helicases play major roles in establishing successful infections of RNA viruses [8]. For example, the helicase domain of the *Brome mosaic virus* (BMV) 1a replicase subunit protein engages in interactions with the 2a^pol^ subunit to target viral replication complexes (VRC) to viral replication sites at the endoplasmic reticulum (ER) [9]. In addition to membrane targeting functions, the C-terminal helicase domain of the BMV 1a subunit functions in recruitment of viral RNA templates to the ER and for efficient replication [9]. An RNA chaperone activity has also been described for the *Tomato bushy stunt virus* (TBSV) p33 replication protein [10] that enables destabilization of the double-stranded RNA replicative form template [11], and provides accessibility of the RNA genome to the viral RNA-dependent RNA polymerase (RdRp) for RNA synthesis [10]. In addition, the cylindrical inclusion (CI) proteins of potyviruses also contain an RNA helicase domain that overlaps the N-terminal and central regions, and functions in RNA replication and cell-to-cell movement [5, 12]. Another strategy used by RNA viruses is to recruit host RNA helicases. In the best studied of these, TBSV recruits multiple host RNA helicases, including DEAD-Box RNA helicases DED1, RH20, RH2, and RH5 to assist in gRNA replication [13–15], and suppression of recombination [16]. In addition, genetic studies of *Turnip mosaic virus* (TuMV) infected *Arabidopsis thaliana* plants have also shown that various host RNA helicases are involved in potyviral infection [17].

The hordeiviruses are positive sense RNA viruses exemplified by the type member, *Barley stripe mosaic virus* (BSMV), which naturally infects barley, wheat and oat, and can also be transmitted to numerous other monocots and dicots by mechanical inoculation [18, 19]. Members of the genus *Hordeivirus* have rigid helical particles that encapsidate tripartite gRNAs designated RNAα, RNAβ, and RNAγ (S2A Fig). The BSMV genome encodes eight major proteins [20–22]. Among these, the αa protein contains highly conserved methyltransferase (MT) and helicase (HEL) domains that function in capping of viral RNAs and unwinding of RNA duplexes during replication and transcription [23]. RNAβ encodes the coat protein, which is responsible for virus particle formation, and the triple-gene block movement proteins (TGB1, TGB2, and TGB3), which are required for intra- and intercellular spread of the virus [24, 25]. RNAγ encodes the γa protein, which has an RdRp activity and interacts with the αa protein to promote RNA replication [26, 27]. Early studies showed that a second ORF translated from the RNAγ subgenomic RNA (sgRNA) encodes a 17 kDa cysteine-rich VSR protein designated γb that has pivotal roles in seed transmission in barley and modulates BSMV symptom severity [28, 29]. In addition, systemic movement in the dicot host *Nicotiana benthamiana* rarely occurs in the absence of γb [30]. Subsequent studies have revealed that γb is a cysteine-rich VSR protein with a zinc finger-like region and protects viral dsRNA from degradation [31, 32]. Four regions in the γb protein are required for maintenance of functional integrity of γb (S2A Fig). Specifically the C1 and C2 regions of γb are responsible for zinc ion binding [33], whereas the basic motif (BM) is critical for nonspecific binding of γb to single-stranded (ss) RNA [34]. The coiled-coil domain in the C-terminal region participates in homologous interaction of γb, which is a prerequisite for RNA silencing suppression activities [31].

Although individual functional studies suggested that γb may have multifunctional roles in viral replication, defined functions of γb remain largely unexplored. Observations of BSMV-infected leaf tissues by transmission electron microscopy have revealed vesicles resulting from invagination of the outer chloroplast membrane [35, 36]. Immunolocalization studies have also shown that double-stranded (ds) RNA is associated with the chloroplast vesicles, but the dsRNA was not specifically shown to be BSMV specific; nevertheless, the vesicles have been hypothesized to be virus replication factories [37]. Consequently, Torrance *et al*. speculated that γb might have unspecified roles in replication because γb was found to localize to chloroplasts in BSMV-infected plants [35].

Our current studies have answered several important questions arising from the previous research. We have provided direct evidence that BSMV ssRNAs and dsRNAs and that BSMV αa and γa are specifically associated with the chloroplasts. We also have found that the αa protein binds directly to the γb protein and facilitates associations of γb with the chloroplasts. To extend these results, we conducted experiments showing that deletion of γb from the BSMV genome resulted in reduced levels of RNAα accumulation in infected *N*. *benthamiana* tissue. We also tested the helicase activity of the use of the αa helicase motif in an *in vitro* cell-free RNA unwinding assay and carried out experiments to test whether αa-γb interactions might affect the αa helicase activity. The results showed that γb alone does not possess helicase activity but suggested that γb greatly enhances αa unwinding of dsRNA duplexes when present in the assays. Overall, these results broaden our understanding of the multifunctional roles of γb, and provide a novel mechanism whereby the γb VSR protein facilitates efficient production of progeny BSMV RNAs.

## Results

### Chloroplasts are major sites of BSMV replication

**Fig 1:**
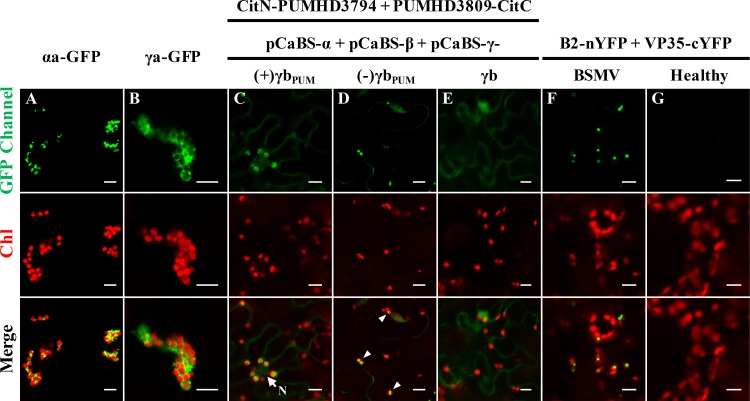
Chloroplast localizations of BSMV replication proteins, single-stranded or double-stranded RNAs. **Panels A and B**: Chloroplast localization of (A) BSMV αa, and (B) BSMV γa. **Note**: Four-week-old systemically infected *N*. *benthamiana* leaves were infiltrated with pSuper1300-αaGFP or pSuper1300-γaGFP and visualized by confocal microscopy at 3 dpi. **Panels C and D**: Chloroplast associations of plus-strand BSMV RNAs (C), and minus-strand BSMV RNAs (D) fused to Pumilio sequences. **Note:** White arrowheads in panel D indicate the chloroplast-associated bright fluorescent puncta. To permit ssRNA visualization, lower leaves were agro-infiltrated with *A*. *tumefaciens* harboring plasmids expressing RNAα or RNAβ, along with RNAγ plasmids expressing the Pumilio PUMHD3794 and PUMHD3809 binding sequences inserted into the positive-sense [(+)γb_PUM_] or negative-sense [(-)γb_PUM_] γb sequence (see S2C Fig). Four weeks afterwards, the upper systemically infected leaves were agroinfiltrated for expression of the split YFP-tagged Pumilio proteins and evaluated by confocal microscopy at 3 dpi. Additional images of the subcellular localization of plus-strand BSMV RNAs [(+)γb_PUM_] are shown in S3A Fig. **Panel E**: Confocal image of a negative ssRNA control with *N*. *benthamiana* infected with wild-type BSMV. **Panel F**: Association of BSMV dsRNAs with chloroplasts. For visualization of dsRNAs, symptomatic leaves of BSMV-infected *N*. *benthamiana* were co-infiltrated with the split YFP-tagged FHV B2 and Marburg virus VP35 proteins, followed by confocal microscopic analysis at 3 dpi. Additional examples of the subcellular localization of BSMV double-stranded RNAs are shown in S3B Fig. **Panel G.** Healthy *N*. *benthamiana* leaves used as a negative control for visualization of dsRNAs. **Note:** *Agrobacterium* derivatives were used for agro-infiltration of *N*. *benthamiana* leaves are indicated above the panels. GFP Channel: confocal microscopy settings: excitation 488 nm, emission 500–530 nm. Chl: chlorophyll autofluorescence. White arrow indicates the nucleus (N). Scale bar, 10 μm.

To determine whether BSMV assembles viral replication complexes on chloroplast membranes, we conducted experiments to test the association of the αa and γa replicase subunits with chloroplasts and the subcellular localization of the BSMV plus- and minus-strand RNAs and dsRNA replicative intermediates. The results at 3 days post-infiltration (dpi) of *N*. *benthamiana* leaf tissues with *Agrobacterium tumefaciens* strains harboring the pSuper1300-αaGFP or pSuper1300-γaGFP plasmid for expression of αa-GFP or γa-GFP respectively, showed that the αa and γa proteins colocalize with chloroplasts (Fig 1A and 1B). We also used a live-cell RNA localization system described by Tilsner *et al*. [38] to visualize the plus- and minus-sense RNAs generated during BSMV replication. In these experiments, a consensus sequence that is specifically recognized by the Pumilio-based reporters was inserted downstream of the γb UAG stop codon in the plus or minus sequence orientations (S2C Fig). These manipulations provided bimolecular fluorescence complementation (BiFC) assays that rely on recruitment of Pumilio split fluorescent protein halves by plus- and minus-sense BSMV RNAs synthesized during replication [38]. The specific binding of the Pumilio proteins to the BSMV RNAγ fragment inserted in either plus or minus sequence orientations enabled detection and localization of BSMV RNAs by confocal microscopy (Fig 1C and 1D). These experiments revealed bright fluorescent puncta representative of plus-sense BSMV RNAs associated with red chloroplast autofluorescence (Fig 1C). Additional fluorescence indicative of plus-strand BSMV RNAs could also be observed as puncta and as diffuse areas in the cytoplasm (S3A Fig). Furthermore, minus-strand RNA foci were detected in association with chloroplasts (Fig 1D, arrowheads). In control experiments, only low background cytoplasmic fluorescence or large nonspecific aggregates were visible in tissue infected with wild type (wt) BSMV lacking the Pumilio recognition sequence (Fig 1E). These results thus confirmed the specific binding of the Pumilio proteins to the engineered BSMV RNA strands.

We also used a dsRNA visualization system developed by Cheng *et al*. [39] to determine the distribution of BSMV-specific dsRNA replication intermediates. In this system, two halves of the yellow fluorescence protein (YFP) were separately fused to two dsRNA binding proteins (B2 of *Flock house virus* and VP35 of Marburg virus), to allow detection of dsRNAs in living cells by reconstruction of the YFP signal [39]. The results of these experiments revealed fluorescent puncta representing the BSMV dsRNAs are intimately associated with chloroplasts in BSMV-infected *N*. *benthamiana* leaves (Fig 1F and S3B Fig). In contrast, fluorescent signals were not evident in the healthy control leaves (Fig 1G). In summary, our results show the colocalization of BSMV replication proteins, plus- and minus-strand BSMV RNAs and BSMV dsRNA intermediates within the chloroplasts, and unambiguously demonstrate that chloroplasts are the major sites of BSMV replication.

### Association of the γb protein with chloroplasts is enhanced during BSMV infection

A previous study indicated that the γb protein localizes to chloroplasts, as well as the cytoplasm during BSMV infection [35]. However, the functional VSR activities of the γb fluorescent protein fusions and their subcellular localization were not tested in these studies. To extend these studies, a spot silencing assay was carried out [40, 41], and the results of this assay indicated that fusion of the GFP and RFP fluorescent proteins to the γb C-terminus had little, if any, effects on RNA silencing suppression (S4 Fig).

**Fig 2:**
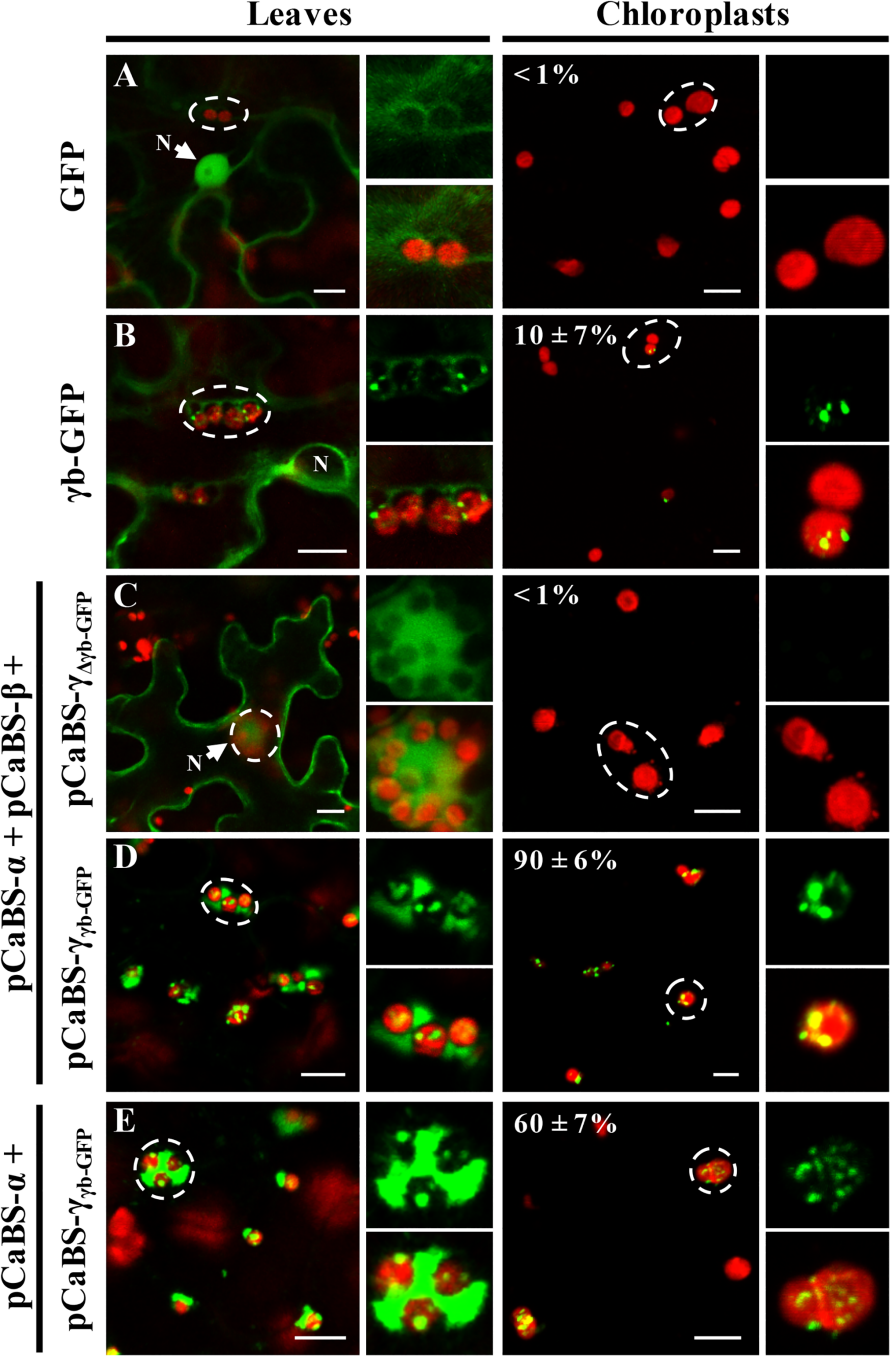
Enhancement of γb chloroplast targeting during BSMV infection. Confocal microscopy images showing green fluorescence and chloroplast autofluorescence in leaf tissue or chloroplasts isolated from the leaves. (**Note**: Leaf tissue and isolated chloroplasts of all panels are designated at the top of the figure. Plasmids indicated to the left of the panels were infiltrated into four-week-old *N*. *benthamiana* leaves for expression of BSMV RNAs and images captured at 3 dpi.). **Panels A and B**: Transiently expressed GFP (A) and γb-GFP (B) at 3 dpi. **Panel C** Fluorescence of BSMV leaves infected by expression of RNAs α + β and γ_Δγb-GFP_. Note: The p19 plasmid was also agroinfiltrated into leaf tissues along with the BSMV plasmids to provide an RNA suppressor function. Also, enlarged images from the RNAs α + β + γ_Δγb-GFP_ agroinfiltrated leaf tissues were taken at a different focal plane from those inside the dashed circle to provide optimal visualization. **Panel D:** Fluorescence in leaves infiltrated with plasmids for expression of RNAs α + β + γ_γb-GFP_. **Panel E:** leaf and chloroplast fluorescence at 3 dpi after expression of RNAs α + γ_γb-GFP_. Regions inside the dashed circles within the low-resolution figures were enlarged and displayed on the right. At least 100 chloroplasts were counted for statistical analysis of each set of infiltrations and the percentages of chloroplasts showing GFP fluorescence are indicated at the upper left corners of the corresponding panels. In panels D and E, lower gain settings were used during confocal analysis of the RNAs α + β + γ_γb-GFP_ or RNAs α + γ_γb-GFP_ infiltrated tissues to highlight γb associations with the chloroplasts. Chl, chlorophyll autofluorescence (in red). White arrows point toward the nuclei (N). Scale bar, 10 μm.

We also used confocal microscopy to determine the subcellular localization of the γb-GFP fusion protein when expressed alone or co-expressed with other BSMV components in BSMV-infected *N*. *benthamiana* leaves. In these experiments, only a low proportion of the γb-GFP protein localized with the chloroplast when only γb-GFP was transiently expressed, as only ~10% of the isolated chloroplasts contained γb-GFP punctata (Fig 2B). In contrast, γb-GFP associated with the ~90% of the isolated chloroplasts in cells infected with RNAs α, β, and γ_γb-GFP,_ and ~60% of γb-GFP fluorescence localized with the chloroplasts isolated from tissue infected RNAs α, and γ_γb-GFP_ (Fig 2D and 2E). In control experiments, the GFP protein localized predominantly in the cytoplasm and also diffused into the nuclei when expressed alone or with other BSMV components (Fig 2A and 2C). Notably, the free GFP fluorescence entered the nucleus because the 27 kDa GFP protein is sufficiently small to diffuse through nuclear pore complexes (Fig 2A and S2B Fig), but the size of the 44 kDa γb-GFP fusion protein restricts passive nuclear diffusion (Fig 2B). Collectively, these results reveal strong associations of γb-GFP with chloroplasts in BSMV-infected tissues and verify that chloroplast associations of γb are greatly enhanced in the context of BSMV infections.

### The γb protein is recruited to the chloroplast through interactions with the αa replicase subunit

**Fig 3:**
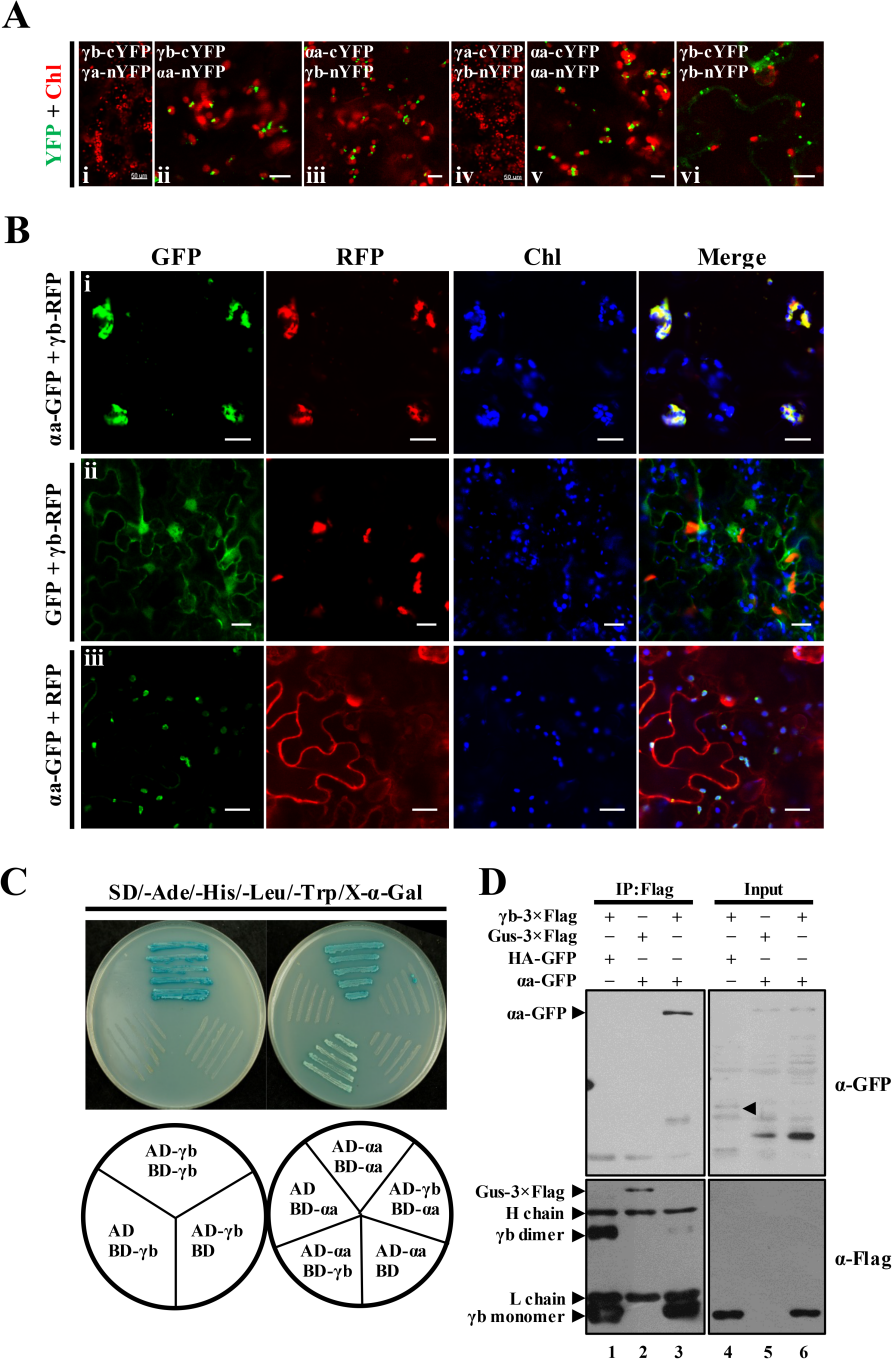
BSMV αa protein mediates recruitment of γb to chloroplasts. **Panel A**. Bimolecular fluorescence complementation (BiFC) analysis of interactions of the γb and αa proteins (**ii** and **iii**) or γb and γa proteins (**i** and **iv**). Combinations of BiFC constructs are shown at the upper left corner of each panel. Chloroplast autofluorescence (Chl) is displayed in red to identify the subcellular localization where the protein–protein interactions occur. Self-interactions of αa or γb are provided as positive controls (**v** and **vi**). Positive YFP signals are depicted as a false-green color. Scale bar, 50 μm for i and iv, and 10 μm for other images. **Panel B**: Confocal microscopic analysis showing that γb-RFP localizes at the chloroplasts in the presence of αa-GFP (**i**), Images showing that expression of the GFP and RFP fusion proteins have little if any effect on the subcellular localization of γb-RFP (**ii**) or αa-GFP (**iii**). Scale bar, 20 μm. **Panel C**. Yeast two-hybrid (Y2H) assays of yeast transformants expressing αa and γb as fusions to the Gal4 activation (AD) or DNA binding (BD) domains. Various combinations of yeast two-hybrid vectors are indicated beneath the corresponding panel. Yeast cells were incubated on yeast synthetic drop-out media (SD-Ade-His-Leu-Trp) supplemented with X-α-Gal. Self-interactions of γb and αa were confirmed by Y2H assays and served as positive controls. **Panel D**: Co-immunoprecipitation (Co-IP) experiments to examine *in vivo* interactions between γb and αa. *N*. *benthamiana* leaf tissues agroinfiltrated with various constructs as indicated in the upper panel were harvested at 3 dpi. Left panels (IP:Flag): total proteins from the harvested samples were subjected to immunoprecipitation with anti-FLAG beads and Western blot analysis using anti-GFP antibody or anti-FLAG antibody. Right panels (Input) shows protein input detected by anti-GFP or anti-FLAG antibodies. Bands corresponding to the target proteins are indicated by arrowheads.

To explore mechanisms whereby γb is targeted to chloroplasts during BSMV infection, BiFC experiments were performed to examine possible interactions between γb and the chloroplast-localized BSMV replication proteins (Fig 1A and 1B). Different combinations (as shown in Fig 3A) of fusion proteins containing N- or C-terminally tagged YFP halves of BSMV αa, γa or γb were tested for possible *in vivo* interactions, which can be observed as reconstructed YFP signals by confocal laser scanning microscopy. The BiFC assay provided convincing evidence that γb interacts with αa in chloroplasts (Fig 3A, ii and iii). In the positive controls, homologous interactions of both αa and γb were observed (Fig 3A, v and vi), in agreement with previous reports [31, 42]. The αa self-interactions were exclusively associated with chloroplasts (Fig 3A, v), whereas γb homologous interactions were largely restricted to the cytoplasm, and the fluorescent puncta typically were distinct from the chloroplasts (Fig 3A, vi). However, interactions between γb and γa were not detected in either BiFC combination (Fig 3A, i and iv). In addition to the BSMV γb-αa interactions, *Lychnis ringspot virus* (LRSV) and *Poa semilatent virus* (PSLV) γb-αa interactions were also observed (S5 Fig). Thus, our results provide strong evidence that similar γb-αa interactions occur during infections of other members of the genus *Hordeivirus*. As a complement to the BiFC assays, total protein samples from *N*. *benthamiana* leaves infiltrated with the BiFC vectors were subjected to Western blot analysis with antibodies against GFP and Actin. The results showed that all half-YFP fusion proteins were expressed in the infiltrated leaf tissues (S6 Fig).

Our results described above suggested that αa might have a direct role in subcellular localization of γb during BSMV infection. To provide additional evidence to support this possibility, transient expression of RFP-tagged γb (γb-RFP) and GFP-tagged αa (αa-GFP) was carried out in *N*. *benthamiana* leaves. The results showed that γb-RFP was redistributed from the cytosol to the chloroplasts when co-expressed with αa-GFP, but remained primarily in the cytoplasm when co-expressed with GFP (Fig 3B, i and ii; Note: Chloroplast autofluorescnce is expressed as a false blue color). In contrast, the chloroplast localization of αa-GFP was not altered during coexpression of γb-RFP (Fig 3B, compare iii with i). These results clearly demonstrate a key role for αa in γb chloroplast targeting.

To further verify αa and γb interactions, yeast two-hybrid (Y2H) and co-immunoprecipitation (co-IP) assays were conducted (Fig 3C and 3D). As a positive control, Y2H analyses confirmed the αa and γb self-interactions suggested by the BiFC experiments (Fig 3A, v and vi). Yeast cells transformed with the αa-activating domain (AD-αa) and the γb-binding domain (BD-γb) plasmids grew well on SD-Trp-Leu-His-Ade drop-out plates as evidenced by the blue color of the streaks (Fig 3C). However, yeast cells with the reciprocal AD-γb and BD-αa plasmids failed to grow on the drop-out plates (Fig 3C), but this may be due to protein topology changes introduced by the γb and αa AD or BD fusions. Yeast cells harboring combinations of the empty vectors (pGBKT7 or pGAD), and the corresponding GAL4 AD or BD fusion constructs failed to grow on the drop-out plates, thus excluding the possibility of auto-activation (Fig 3C). In addition, interactions between αa and γb were also revealed by immuno-precipitation (IP) assays (Fig 3D). The γb-3×Flag and αa-GFP fusions were transiently expressed via agro-infiltration under control of the 35S and Super promoters, respectively. Total proteins were solubilized in GTEN buffer containing 1% Triton X-100 and 0.15% NP-40, and subjected to IP assays with FLAG-beads. In these experiments, αa-GFP co-precipitated with γb-3×Flag (Fig 3D, lane 3), whereas appreciable GFP-fusion proteins were not detected in negative controls when either γb-3×Flag was replaced by GUS-3×Flag, or αa-GFP was replaced by HA-GFP (Fig 3D, lanes 1–2). Taken together, these results demonstrate that αa engages in specific physical interaction with γb, and that αa-γb interactions result in the recruitment of γb to chloroplasts.

### Mapping key regions that determine interactions between γb and αa

**Fig 4:**
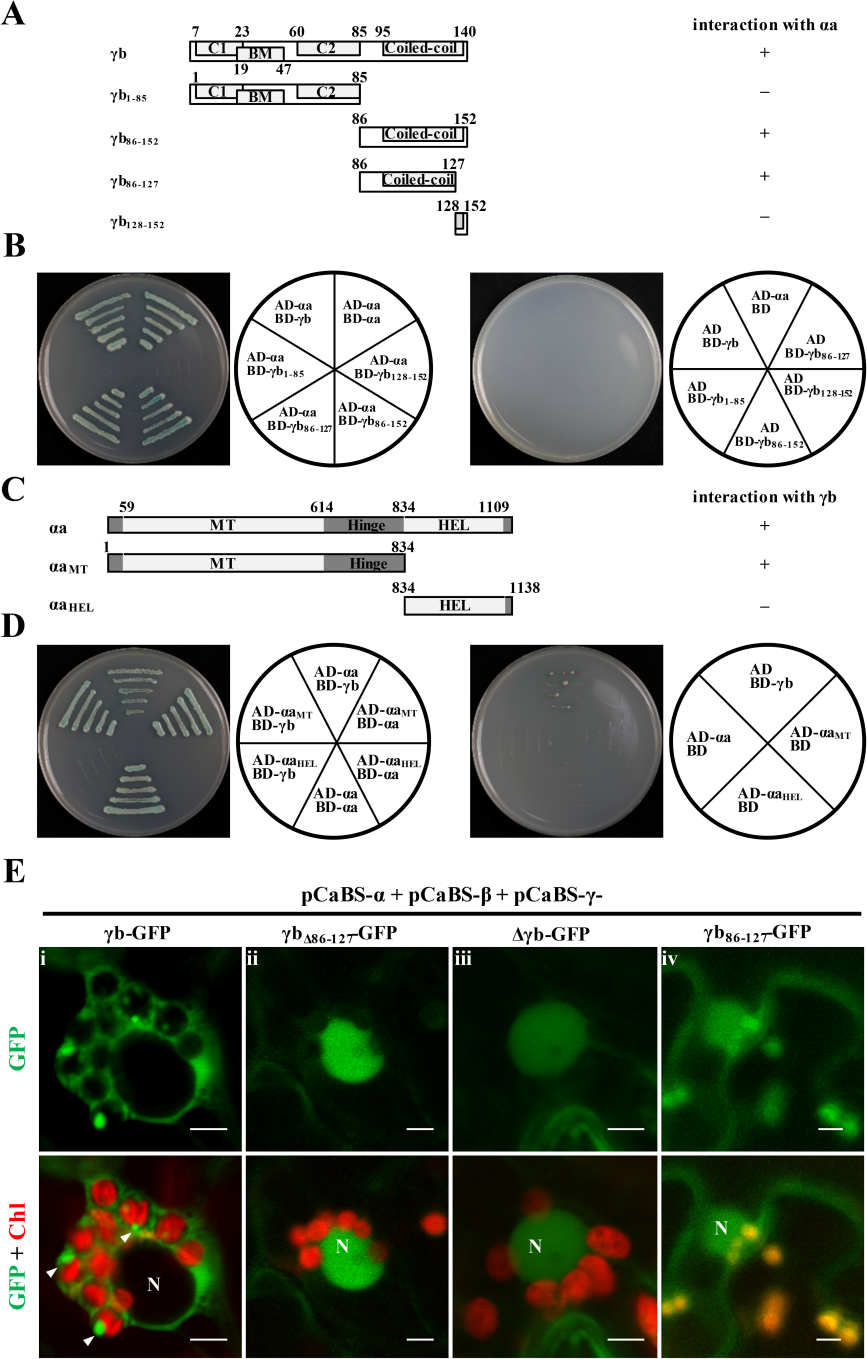
Yeast two-hybrid mapping of γb and αa protein interacting regions. **Panel A**: Schematic representation of γb truncation or deletion mutants used for Y2H assays. Interactions with αa are indicated on the right. **Panel B**: Y2H mapping of regions within γb that interacted with αa. Various γb mutants were cloned into the bait vector pGBKT7 and tested for interactions with the αa protein expressed from the prey vector pGADT7. The divided circles illustrate different vector combinations. **Panel C**: Schematic representation and interactions (right side) of αa truncation or deletion mutants tested for interactions with γb in Y2H assays. **Panel D**: Y2H mapping of αa regions that interact with γb. In Panels A and C, “+” indicates γb or αa regions that are involved in γb-αa interaction, whereas “–” indicates regions that are not involved in γb-αa interactions. In Panels B and D, yeast cells were co-transformed with the prey and bait plasmids as depicted in the diagrams to the right of each panel, and incubated on yeast synthetic drop-out media (SD-Ade-His-Leu-Trp) supplemented with X-α-Gal. Combinations of γb or αa derived bait vectors with the empty prey vector pGADT7, or the empty bait vector pGBKT7 with the αa or γb derived prey vectors, served as negative controls. **Panel E:** Subcellular localization of γb deletion mutants at 3 dpi. *Agrobacteria* containing pCaBS-α, pCaBS-β and various pCaBS-γ derivatives shown in S2C Fig were agroinfiltrated into *N*. *benthamiana* leaves, followed by confocal microscopy analyses. Different pCaBS-γ derivatives used for agroinfiltration are indicated (**i**-**iv**). Chlorophyll autofluorescence (Chl) is shown in red. Nuclei are identified by N. White arrowheads indicate fluorescent aggregates associated with the chloroplasts. Scale bar, 5 μm.

To identify which regions of γb and αa are responsible for γb-αa interactions, we constructed a series of γb and αa deletion mutants for yeast two-hybrid assays (Fig 4A and 4C). Fragments of γb were expressed as fusion proteins with the GAL4 DNA-binding domain and tested for their possible interactions with full-length αa fused with the GAL4 activation domain (AD-αa). In these tests, yeast expressing the BD-γb_86-152_ containing C-terminal 86–152 amino acids (aa) of γb grew well on Trp, Leu, His, and Ade drop-out plates, whereas yeast expressing BD-γb_1-85_ failed to grow (Fig 4B, left panel). Truncations of the γb C-terminal sequences further indicated that γb amino acids 86–127 retained the ability to interact with αa, whereas γb_128-152_ was unable to participate in γb-αa binding (Fig 4B, left panel). These data indicate that the γb C-terminal region between amino acids 86 and 127 is required for γb-αa interactions.

We also mapped the regions of αa that interact with γb by constructing GAL4AD fusions to different αa truncations (Fig 4C) and tested for interactions with the BD-γb. Y2H analysis showed that methyltransferase (MT) with hinge-like domain was able to participate in γb-αa binding, whereas the C-terminal helicase (HEL) domain was unable to interact with γb (Fig 4D, left panel). As negative controls, various constructs harboring the αa and γb-derived mutants were co-transformed with empty yeast vectors. The results show that these controls are unable to grow on dropout medium lacking Ade, Trp, Leu, and His (Fig 4B and 4D, right panels).

We next conducted experiments to assess whether the mapped γb region responsible for interactions of γb with αa contributes to chloroplast targeting of γb during BSMV infection. The plasmid pCaBS-γ_γb-GFP_ reporter construct was mutagenized to retain or eliminate γb residues 86–127 that constitute the γb-αa interaction region (S2C Fig). *N*. *benthamiana* leaves were coinfiltrated with *Agrobacterium* strains containing pCaBS-α, pCaBS-β, and individual pCaBS-γ_γb-GFP_ derivatives to assess γb-GFP subcellular localization during infection. In comparison with wt γb-GFP, which formed fluorescent rings around the chloroplasts, and fluorescent puncta on the chloroplasts (Fig 4E, i, Note arrowheads), deletion of the αa-interacting region within γb (aa 86–127) resulted in reduced chloroplast associated fluorescence (Fig 4E, ii, γb_Δ86-127_-GFP) that were similar to the GFP substitutions for the γb ORF in RNAγ (Fig 4E, iii). In contrast, the αa-interacting region of γb-GFP alone (aa 86–127) was able to localize to the chloroplasts during BSMV replication (Fig 4E, iv). These results thus provide persuasive evidence that physical interactions of γb residues 86 to 127 are required to mediate αa binding and recruitment of γb to chloroplasts.

### The γb protein is required for efficient accumulation of BSMV plus-strand RNAα

Because RNAβ encodes the coat protein and the triple gene block proteins that are essential for virion formation and movement (S2A Fig), these processes could interfere with studies of RNA replication *per se*. Therefore, to avoid these problems, we eliminated pCaBS-β for the experiments below and infiltrated *N*. *benthamiana* leaves with *Agrobacterium* strains harboring pCaBS-α and various pCaBS-γ mutant derivatives. This procedure eliminated BSMV cell-to-cell movement and ensured that replication was restricted only to the initially infected cells, and that virion morphogenesis and movement processes did not affect BSMV RNA accumulation. Moreover, the high percentages of leaf cells transiently expressing BSMV RNA and proteins after agroinfiltration [43], resulted a high proportion of infected cells in the infiltrated leaf tissue, while also enhancing the likelihood that infected cells were undergoing synchronous replication at 3 dpi.

One question arising from our results is whether the γb protein participates directly in the replication of BSMV RNAs, or if the effects are due to an RNA segment within the γb ORF. To address this issue, we generated two mutants in the RNAγ infectious clone (pCaBS-γ) to differently affect γb protein or RNA synthesis. The first mutant (designated γ_Δγb_) was engineered by deleting the γb ORF in order to eliminate both γb protein and RNA synthesis, and the second mutation (γ_γbATGm_), generated to maintain the γb ORF RNA sequence, was constructed by introducing a single site specific mutation (AUG→UUG) into the γb start codon (S2C Fig).

**Fig 5:**
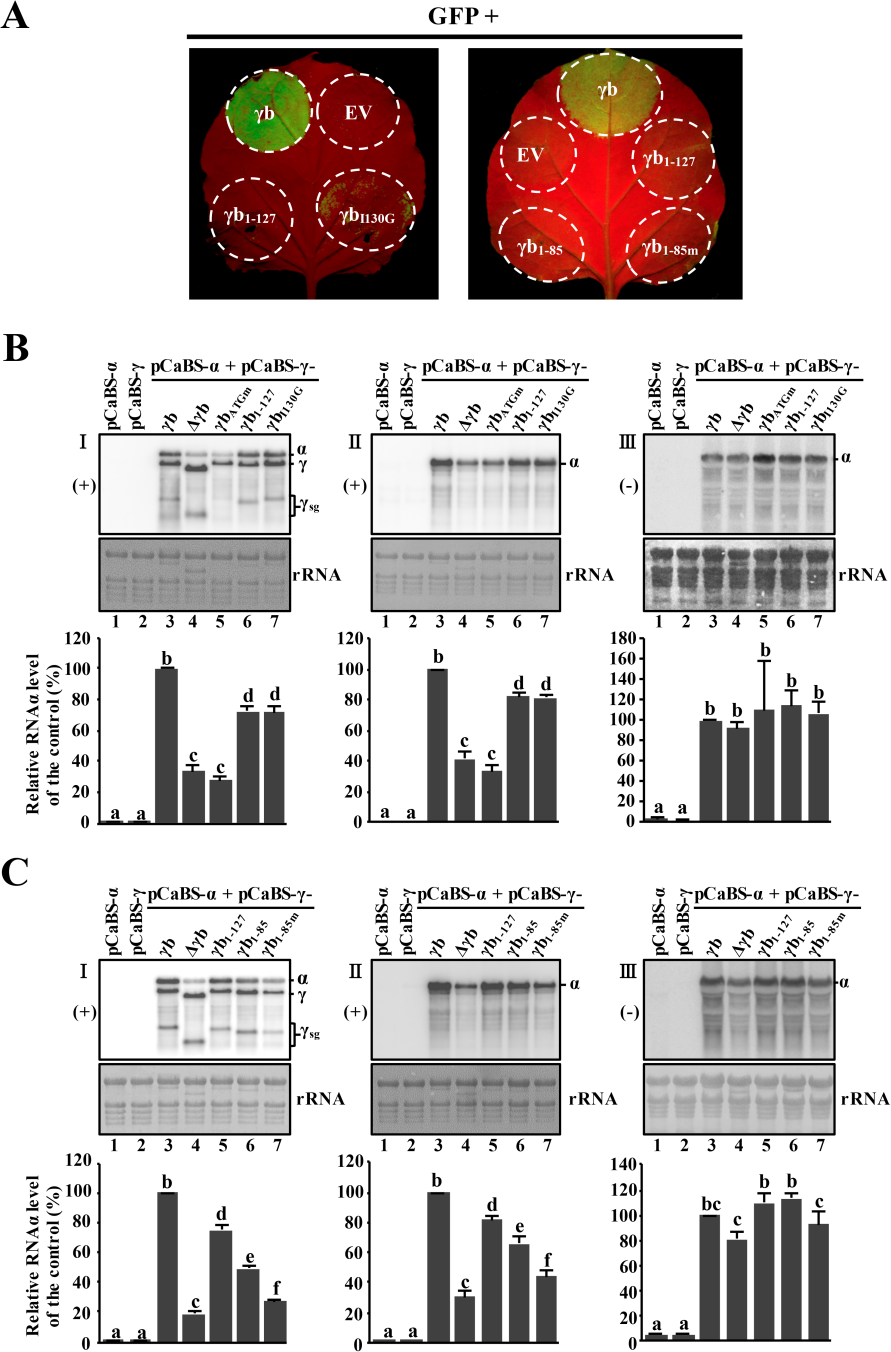
Multifunctional roles of γb in accumulation of BSMV RNAs. **Panel A**: RNA silencing suppressor activities of different γb mutants. Circles in the photos indicate regions of *N*. *benthamiana* leaves infiltrated with *A*. *tumefaciens* harboring plasmids expressing γb derivatives and GFP. Regions infiltrated with wild-type γb or the empty pGD vector (EV) served as positive or negative controls. Leaves were observed at 3 dpi under UV illumination. **Panels B and C**: Northern blot analyses of BSMV RNAs isolated from infiltrated tissue. *N*. *benthamiana* leaf tissues were agroinfiltrated with pCaBS-α and/or various pCaBS-γ derivatives shown in S2C Fig. Combinations of vector constructs used for Northern blot assays are indicated at the top of each lane. At 3 dpi, total RNA was isolated from the inoculated leaves and subjected to Northern blot analysis. **Note:** The left and middle series (**I** and **II**) of images in Panels B and C depict plus-strand RNAs and images on the right side (**III**) show minus-strand RNAs. BSMV plus-strand (+) RNAs were detected with the 3′-UTR probe, and plus-strand (+) RNAα was visualized with the plus-strand RNAα specific probe. Minus-strand (-) RNAα was detected with the minus-strand RNAα probe. Bands corresponding to RNAα, RNAγ and subgenomic (sg) RNAγ are indicated along the right sides of the northern blots. Note: Due to reasons that have yet to be identified, RNAγ_γbATGm_ expressing tissues consistently accumulated lower levels of subgenomic RNA (γ_sg_) when co-inoculated with RNAα than tissue RNAα + RNAγ_Δγb_. Methylene blue stained ribosomal RNAs were used to show equal loading. Intensities of the RNAα-specific bands were measured, normalized and analyzed statistically using procedures described in the Materials and Methods. Quantifications of RNAα accumulation from different lanes in panels B and C are shown in the bar graphs. The graphic data represent the means of three independent experiments. Error bars indicate standard error (n = 3). In each bar chart, different letters above bars denote statistically significant differences according to the Duncan's multiple range test (*P* < 0.05). In Panel C, lane 6 shows analysis of BSMV RNA accumulation in leaves expressing the γb_1-85_ protein in which the αa-interacting regions had been deleted, and lane 7 shows RNA accumulation in tissue expressing γb_1-85m_ protein in which the RNA binding activities have been eliminated.

We co-infiltrated BSMV RNAα and the RNAγ derivatives to initiate BSMV replication. At 3 dpi, total RNA from the infiltrated regions was extracted and subjected to Northern blot analysis by using the 3′-untraslated region (UTR) probe or the RNAα-specific probe to monitor accumulation of BSMV RNAs. Due to the high nucleotide sequence identity amongst the 3′-UTR regions of the genomic RNAs [44], accumulation of α and γ plus-strand RNAs and sgRNAγ could be detected simultaneously. The RNAα-specific probe enabled specific detection of the RNAα plus-strand, and a BSMV RNAα probe for detection of minus-strand RNAα was also used. As expected, *N*. *benthamiana* leaves infiltrated with pCaBS-α or pCaBS-γ alone resulted in barely detectable RNA accumulation at 3 dpi (Fig 5B and 5C; panels I and II, lanes 1 and 2), suggesting that BSMV was not replicating in the infiltrated tissue. In contrast, high-levels of wt BSMV gRNAs and sgRNAγ accumulation were detected at 3 dpi after co-infiltrations of pCaBS-α and pCaBS-γ (Fig 5B and 5C, panels I and II, lane 3). However, co-infiltrations with pCaBS-α and pCaBS-γ_Δγb_, in which the γb ORF had been deleted, or pCaBS-α and pCaBS-γ_γbATGm_, which only eliminated γb AUG codon, both resulted in substantially impaired accumulation of BSMV plus-strand RNAα (Fig 5B, panels I and II, compare lanes 4 and 5 with lane 3), but not the minus-strand RNAα (Fig 5B and 5C, panel III; compare lane 4 or 5 with lane 3). These results thus strongly implicate γb protein functions in accumulation of plus-strand RNAα. Nevertheless, accumulation of RNAγ appeared not to be substantially affected by the γb deletion/mutations. This suggests a model whereby replication of RNAα, but not RNAγ, is *cis*-preferential [27], and that γb-αa protein interactions are required for efficient *cis*-preferential replication of the plus-strand of RNAα (see discussion).

To provide additional information about the effects of γb on BSMV infection, other BSMV mutants were constructed in pCaBS-γ. These consist of RNAγ_γb-GFP_, which contained an N-terminal GFP fusion, a truncated γb derivative, (RNAγ_γb1-127-GFP_) designed to eliminate suppression of RNA silencing and a null mutant (RNAγ_Δγb-GFP_) generated by substitution of the *gfp* ORF for the γb ORF (S7A Fig). These RNAγ derivatives were agroinfiltrated into plant leaves along with wt RNAα (S7B Fig), and the results at 3 dpi showed that γb_1-127_-GFP, which has a deletion of 25 amino acids from the γb C-terminus and lacks VSR activity, had much lower amounts of GFP in the infiltrated regions than the full length γb-GFP protein and the amounts of GFP in the null mutant regions were even lower than those γb_1-127_-GFP (S7C and S7D Fig). These results thus provide additional evidence that, in addition to its VSR activities, γb has other functions that participate in efficient BSMV replication.

A previous report also showed that deletion of the γb ORF from RNAγ affected BSMV pathogenesis in barley [29], but these experiments did not determine directly whether deletion of the sequences affected BSMV pathogenesis in *N*. *benthamiana*. Therefore, we co-infiltrated pCaBS-α and pCaBS-β along with pCaBS-γ, or the pCaBS-γ mutants γb_ATGm_, or Δγb to determine whether presence of the RNA sequence encoding the γb protein could ameliorate the pathogenic effects that occur when the γb ORF is deleted. Symptoms arising from both the γb_ATGm_ and the Δγb mutant co-infiltrations elicited very mild symptoms in infected *N*. *benthamiana* (S8A Fig) and also destroyed γb expression (S8B and S8D Fig). Systemic BSMV symptoms in barley also were severely affected by both mutants (S8C Fig), and resulted in the “Null” chlorotic streak phenotype previously reported when the γb ORF was deleted from RNAγ [29]. In contrast, expression of wtRNAγ in combination with RNAβ and RNAγ resulted in typical chlorotic or mosaic symptoms in systemically infected *N*. *benthamiana* and barley leaves and nearly 100% of the inoculated plants were infected (S8A and S8C Fig). Thus, our results verify that the BSMV wild-type phenotypic effects in both barley and *N*. *benthamiana* depend on expression of the γb protein, and that sequences within the γb ORF in RNAγ can not compensate for the absence of γb.

Considering that γb is a multifunctional protein [29, 31, 34], we extended the analyses to more clearly define γb mutant activities that inhibit replication of RNAα. Previous studies indicated that arginine and lysine residues at amino acid (aa) residues 25 and 26 in the γb basic motif of γb are essential for single-strand RNA binding activity [34], and that seven residues within positions 102 to 130 in the C-terminal region of the γb protein are critical for maintenance of RNA silencing suppressor activity [31]. Therefore, two γb mutants similar to those previously reported [31] were introduced into pCaBS-γ to determine their effects on silencing suppressor activities. Specifically, these mutants included γb_1-127_, whose product contains a C-terminal 25 aa residue truncation, and γb_1-85_, which contains a 67 aa truncated product that lacks the αa-interacting region present within γb_1-127_ (S2B and S2C Fig), and largely maintains the RNA binding activity [45]. Addition mutants included γb_1-85m_, to produce substitutions (^25^RK^26^ to ^25^QN^26^) of adjacent basic motif residues in the γb_1-85_ protein that affect RNA binding [29, 31, 34]. We also constructed a single substitution mutant (γb_I130G_) whose product contains a Gly residue at the end of the coiled-coil motif that inhibits RNA silencing suppressor activities of the wt γb protein (S2B and S2C Fig). The results of spot silencing experiments with these γb derivatives showed that the γb_1-85_, γb_1-85m_ and γb_1-127_ proteins completely failed to exhibit RNA silencing suppressor activity, and that the γb_I130G_ γb point mutant protein resulted in greatly reduced levels of RNA silencing suppressor activity (Fig 5A).

In addition, the effects of the γb mutations on BSMV replication were tested by Northern blotting. Control comparisons revealed that plus-strand RNAα accumulation in the RNAα + RNAγ infiltrations was more than two-fold greater than those of the RNAα + RNAγ_Δγb_ infiltrations (Fig 5B, panels I and II, lanes 3 and 4,). The results also indicated that accumulation of plus-strand RNAα in plants with BSMV expressing γb_1-127_ and the γb_I130G_ mutants was ~70% of the level of the wt γb control (Fig 5B, panels I and II, compare lanes 6 and 7 with lane 3). In addition, plus-strand RNAα accumulation in infections with the γb_1-127_ and the γb_I130G_ mutants (both of which eliminate suppression of RNA silencing) was more than two-fold higher than RNAα accumulation in the RNAα + RNAγ_Δγb_ or RNAα + RNAγ_γbATGm_ infections (Fig 5B, panels I and II, compare lanes 6 and 7 with lanes 4 and 5). The results also show that the mutants do not have substantial effects on the accumulation of minus-strand RNAα (Fig 5B, panel III). Thus, these results suggest that regions within the γb N-terminal 127 aa may have a role in RNAα accumulation, and that γb RNA silencing suppressor activity *per se* is not entirely responsible for efficient replication of BSMV plus-strand RNAα.

Next, we compared wt γb and RNAγ_γb1-127_ with more extensive γb C-terminal deletion mutants (RNAγ_γb1-85_, and RNAγ_γb1-85m_) that eliminate the αa interacting region in γb (Fig 5C, panels I and II). Comparisons with RNAγ_γb1-127_ revealed that plus-strand RNAα accumulation was nearly 70% of wt γb control and that RNAα accumulation in RNAγ_γb1-85_ was at least 50% of wt γb (Fig 5C, panels I and II, compare lanes 3, 5 and 6). Again, none of the mutants had substantial effects on the accumulation of minus- strand RNAα (Fig 5C, panel III). These results suggest that γb interactions with αa are important for maintenance of the normal accumulation of viral plus-strand RNA during BSMV replication. In separate experiments, when the BM motif (^25^RK^26^→^25^QN^26^), which is involved in RNA binding, was mutated in γb1-85 (γb_1-85m_), the plus-strand RNAα levels decreased to about one-half of that in RNAα + RNAγ_γb1-85_ and were nearly the same levels as in RNAα + RNAγ_Δγb_ infected cells (Fig 5C, panels I and II, compare lanes 4 and 6 with lane 7). Taken together, these results suggest that multifunctional activities of the γb protein affect RNAα accumulation. The γb RNA silencing suppressor activity has a limited positive effect on RNAα replication and additional γb functions involving γb-αa and RNA binding activities may interact synergistically in accumulation of BSMV RNAα.

### The p19 VSR protein fails to rescue plus-strand RNAα replication of γb-deficient BSMV mutants

**Fig 6:**
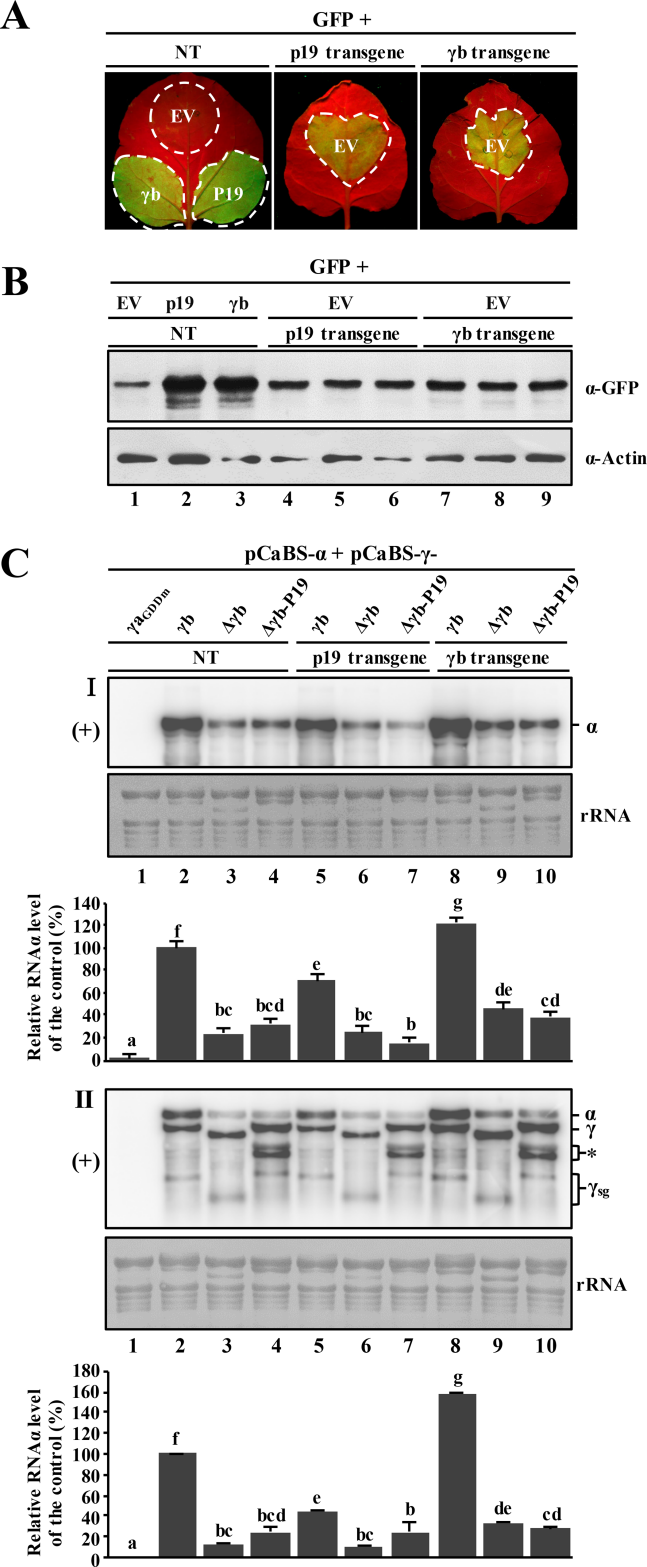
Inability of the p19 VSR protein to restore RNAα accumulation in leaves infected with γb-deficient BSMV mutants. **Panel A**: Spot silencing assays comparing RNA silencing suppression abilities of transgenic *N*. *benthamiana* expressing p19 or γb versus non-transgenic *N*. *benthamiana*. The plants were co-infiltrated with the 35S-GFP vector plus the pGD empty vector (EV), and observed at 3 dpi under UV illumination for GFP fluorescence. Transient expression of γb in non-transgenic plants served as positive controls. **Panel B:** Western blot analysis of GFP expression in agro-infiltrated regions of the *N*. *benthamiana* leaves shown in Fig 6A. Sample loading was determined by total Actin immunoblots. **Panel C:** Northern blot analysis of viral plus-strand (+) RNA accumulation in γb-deficient BSMV infected leaves with RNAα- (panel I) and 3′-UTR-specific (panel II) probes, respectively. The p19 or γb expressing transgenic plants or non-transgenic plant leaves were agroinfiltrated with the RNAα + RNAγ-derivatives as shown above the panel lanes. The γa replicase was inactivated by mutating the GDD motif to GAD (γa_GDDm_) in order to prevent BSMV replication. RNAα + RNAγ_γaGDDm_ served as a negative control (lane 1). Information about the plasmids used for infiltration is shown in S2C Fig. RNAα, RNAγ and sgRNAγ bands are indicated on the right. **Note**: the doublets migrating between RNAγ and γ_sg_ (panel II, asterisk) have an unknown origin and were observed in previous studies [92]. Methylene blue stained rRNAs were used as a loading control. Blots are representative of three independent experiments. Comparisons of RNAα levels were shown below the corresponding rRNA loading panel. The bar graphs below the RNA blots show the means of three independent experiments. Error bars indicate standard error (n = 3). In each bar chart, different letters above the bars denote statistically significant differences (*P* < 0.05) determined by the Duncan's multiple range test.

To determine whether the compromised γb VSR activity in the γb-deficient BSMV mutants can be complemented by other known VSRs, we expressed the TBSV p19 protein, a well-studied RNA silencing suppressor [46, 47], *in cis* and *in trans* to investigate whether this heterologous VSR protein can rescue BSMV replication defects elicited by the γb mutants. First, we cultivated transgenic *N*. *benthamiana* plants, and conducted genomic PCR analysis to verify γb or p19 transformation. The results of growth experiments revealed that compared to non-transgenic plants, the p19-transgenic plants have a stunted growth phenotype as well as mild leaf curling (S9A Fig), and PCR experiments showed that all of the tested transgenic plants were positive for genomic γb or p19 DNA integration (S9B Fig). In addition, spot silencing experiments indicated that the RNA silencing suppression activity by γb- and p19-transgenic *N*. *benthamiana* were both moderate as indicated by comparisons of the GFP fluorescence of infiltrated leaves with agrobacterium strains harboring γb or p19 (Fig 6A). However, the suppressor activity in the transgenic leaves was much more pronounced than with agrobacterium control infiltrations with the empty vector (EV) plasmid (Fig 6A). Western blot analysis of protein extracts from agro-infiltrated leaf areas also confirmed the *gfp* silencing phenotypes observed under UV light (Fig 6B).

Next, *Agrobacterium* strains for expression of RNAα together with individual RNAγ derivatives shown in S2C Fig were co-infiltrated into leaves of non-transgenic, p19-transgenic and γb-transgenic *N*. *benthamiana* plants, respectively. Amongst the infiltrated leaves, RNAγ with an amino acid mutation in the GDD motif (GDD→GAD) that abolishes RdRp activity of γa served as a negative control. At 3 dpi, total RNA was extracted from agro-infiltrated leaves followed by Northern blot analyses with either the RNAα- or 3′-UTR-specific probe. As shown in Fig 6C, the RNAα plus RNAγ_γaGDDm_ agroinfiltrations had undetectable levels of the BSMV RNAs (Fig 6C, panels I and II, lane 1), whereas in the RNAα + RNAγ positive controls, highly abundant plus-strand RNAs could be detected in both the transgenic and non-transgenic *N*. *benthamiana* plants (Fig 6C, panels I and II, lanes 2, 5 and 8). The non-transgenic *N*. *benthamiana* leaf discs that had been agroinfiltrated to express RNAα + RNAγ_Δγb_ had significantly reduced levels of BSMV replication compared to the RNAα + RNAγ controls (Fig 6C, panels I and II, lane 3), in agreement with the results shown in Fig 5. In contrast, when TBSV p19 was expressed *in cis* by replacing the γb ORF in RNAγ (γ_Δγb-P19_), the BSMV plus-strand RNA signaling intensity was similar to that of the γb-deficient (RNAα + RNAγ_Δγb_) infiltrated tissue (Fig 6C, panels I and II, compare lane 3 with 4). Similar trends were also evident when these BSMV derivatives were agroinfiltrated into p19-transgenic *N*. *benthamiana* (Fig 6C, panels I and II, lanes 6 and 7). Compared to viral replication in non-transgenic plants, expression of the p19 transgene also failed to enhance the accumulation of either the γb-deficient virus (RNAα + RNAγ_Δγb_) or accumulation of RNAα + RNAγ_Δγb-P19_ (Fig 6C, panels I and II, compare lanes 6 and 7 with lane 2). Surprisingly, replication of the wt RNAα + RNAγ controls was moderately inhibited in p19-transgenic *N*. *benthamiana* (Fig 6C, panels I and II, compare lane 2 with lane 5). In contrast, when the wt BSMV derivatives were agroinfiltrated into γb-transgenic *N*. *benthamiana*, RNAα replication was augmented to varying degrees, especially in the wt RNAα + RNAγ control, whose RNAα accumulation increased by more than 140 percent over the non-transgenic control (Fig 6C, panels I and II, compare lane 8 with lane 2). The replication of BSMV RNAs in leaf tissue agroinfiltrated with both RNAα + RNAγ_Δγb_ and RNAα + RNAγ_Δγb-P19_ increased only moderately with overexpression of γb *in trans* in the γb-transgenic plants, and did not reach the same levels as those of the wt RNAα + RNAγ infiltrations (Fig 6C, panels I and II, compare lanes 9–10 with lanes 2–4). Hence, these results show that *trans* expression of γb or p19 in transgenic plants failed to rescue the reduced plus-strand RNAα accumulation observed in infections with RNAα + RNAγ_Δγb_.

In an additional approach to determine whether ectopic expression of γb from subgenomic RNAβ via replication could complement the γb functions during BSMV replication, we replaced the RNAβ TGB1 ORF with the γb ORF (S10A Fig). However, despite efficient expression of γb from the modified RNAβ (S10B Fig, panel I, lanes 5–6), enhancement of RNAα replication by γb was not evident (S10B Fig, panels III and IV, compare lanes 5 and 6 with lane 2). These results suggest that the ability of γb to promote RNAα replication requires strict temporal and spatial expression of γb during BSMV infection from sgRNAγ. Our results also indicate that expression of HCPro-transgenic *N*. *benthamiana* does not rescue replication defects exhibited by γb-deficient BSMV mutants (S11 Fig). These results differ somewhat from those of Yelina *et al*. [30], who reported that systemic movement of γb-deficient BSMV was recovered in HCPro-transgenic *N*. *benthamiana*. Taken together, our experiments show that *in trans* expression of γb, p19 or HCPro does not fully complement the γb deletion phenotype, and that fully functional γb requires expression *in cis* from sgRNAγ. Moreover, *in cis* expression of p19 from the sgRNA also failed to complement the γb deletion phenotype, indicating that optimal BSMV replication requires multiple γb functions in addition to those directed towards suppressor activities *per se*.

### γb RNA binding activities provide an enhancer role in unwinding of RNA duplexes by the αa helicase

**Fig 7:**
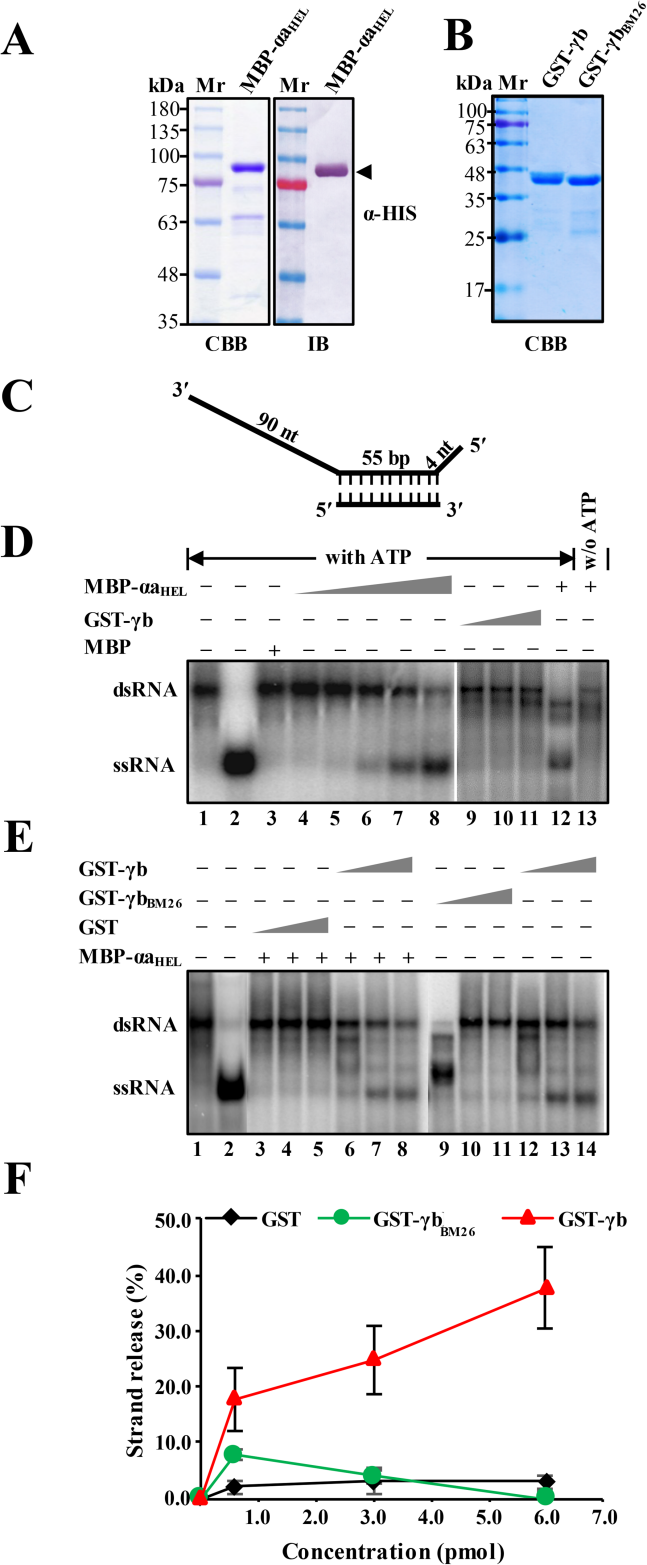
Enhancement of αa_HEL_ protein RNA duplex unwinding by the γb protein. **Panel A**: SDS-PAGE and immunoblot analysis of purified MBP-αa_HEL_ protein. The recombinant MBP-αa_HEL_ protein containing a histidine tag and the αa helicase domain fused to the C-terminus of the maltose binding protein was expressed in *E*. *coli* strain BL21 and purified over Ni−NTA agarose columns. SDS-PAGE staining with Coomassie brilliant blue (CBB) is shown on the left side and immunoblot analysis (IB) with α-HIS antibodies is shown on the right side. **Panel B:** SDS-PAGE analysis of GST-tagged γb derivatives purified from *E*. *coli*. Sizes (in kDa) of molecular weight markers (Mr) are shown on the left sides of the blots. **Panel C**: Schematic depiction of the dsRNA substrate used for RNA unwinding assays. Preparation of the radioactive labeled substrate is described in the Materials and Methods and in S2 Table. The long and short thick lines indicate the template RNA strands, and vertical dashed lines represent regions of base pairing. The lengths of the double-stranded and overhanging portions of the RNA strands are given in base pairs (bp) and nucleotides (nt), respectively. **Panel D.** RNA helicase activities of the purified MBP-αa_HEL_ and GST-γb proteins in the presence or absence of ATP. The mobility of the radioactive labeled RNAs was tested using native acrylamide gel electrophoresis followed by phosphor imaging. Lane 1, migration of the partial dsRNA band. Lane 2, migration of single-stranded RNA produced by heating the dsRNA substrate at 95°C for 5 min. Lane 3, Negative control showing lack of helicase activity of purified MBP. Lanes 4–8, RNA unwinding reactions containing increasing amounts of purified MBP-αa_HEL_ (1.8–6.2 pmol) in the presence of 5 mM ATP. Lanes 9–11, RNA unwinding reactions containing increasing amounts of purified GST-γb (1.8–6.2 pmol) in the presence of 5 mM ATP. Lanes 12–13 indicate the RNA helicase activity of MBP-αa_HEL_ with ATP or without (w/o) addition of ATP to the reaction mixture. **Note:** Lanes 1–8 and 9–13 were separated on separate gels. **Panel E.** dsRNA unwinding of MBP-αa_HEL_ in RNA helicase reactions containing wt GST-γb, GST-γb_BM26_ or GST. Helicase assays were carried out with 4.4 pmol of the MBP-αa_HEL_ protein in the presence of increasing concentrations (0.5, 3.0, 6.0 pM) of GST (lanes 3–5), γb (lanes 6–8, 12–14) or γb_BM26_ (lanes 9–11) respectively. Positions of the partial dsRNA substrate and the released 55 nt ^32^P ssRNA strand product are indicated on the left (lanes 1–2). **Note:** Lanes 1–8 and 9–14 were separated on separate gels. **Panel F:** Quantification of dsRNA unwinding efficiency shown in Fig 7E. The results are plotted as percentages of ssRNA release compared to the ssRNA in lane 2.

Based on Fig 5C showing the replication of RNAα in infected tissue elicited by RNAα + mutant RNAγ_1-85m_ containing two basic motif mutations (^25^RK^26^→^25^QN^26^), and on a previous *in vitro* binding assay in which a full-length mutant γb protein containing the same substitutions failed to bind ssRNA [34], we hypothesized that γb might have functions related to the single-stranded DNA-binding proteins (SSBs) that participate in DNA replication as helicase enhancers [48]. To test this hypothesis, we constructed an *E*. *coli* plasmid (pDB.His.MBP-αa_HEL_) for expression of a maltose binding protein (MPB) fused to αa amino acids 614–1138, which comprise the C-terminal helicase domain (S2A Fig). We then used the plasmid for *E*. *coli* expression, affinity purification, SDS-PAGE and Western blots of the MBP-αa_HEL_ recombinant protein. The results confirmed high level purification of the MBP-αa_HEL_ protein with only traces of *E*. *coli* derived proteins (Fig 7A). We also used pDB.His.MBP for construction and purification of a GST-tagged wt γb protein (GST-γb), and a GST-tagged γb mutant protein (GST-γb_BM26_) that is defective in RNA binding (Fig 7B).

To evaluate the roles of the recombinant proteins in RNA duplex unwinding, we designed an *in vitro* helicase assay consisting of a partially RNA duplex substrate, which provided a 55 nt dsRNA base paired region flanked by a 90 nt single-stranded 3′-sequence and a short 4 nt ssRNA 5′-overhang (Fig 7C). This structure enabled testing of the helicase activity of the recombinant proteins to assess their abilities to release a radiolabeled 55 nt single strand RNA from the duplex substrate.

Addition of the MBP-αa_HEL_ fusion protein in amounts ranging from 1.8 to 6.2 pmol to a helicase mixture containing ATP and the radioactive duplex substrate resulted in release of increasing amounts of ssRNA (Fig 7D, lanes 4–8). However, when ATP was omitted from reactions containing 8.9 pmol of the MBP-αa_HEL_ fusion protein, single strand RNA was not released (Fig 7D, compare lane 12 with lane 13). The results also show that the MPB-αa_HEL_ helicase activity is dosage dependent, as the lower MBP-αa_HEL_ concentrations (< 4.4 pmol) failed to release substantial amounts of labeled 55 nt RNA from the 0.32 pmol dsRNA duplex. Control experiments indicated that the helicase activity is specific to the MBP-αa_HEL_ protein rather than MBP or minor contaminating *E*. *coli* proteins because > 6 pmol of MBP purified from pDB.His.MBP in reactions containing ATP failed to release the radiolabeled 55 nt RNA strand from the duplex substrate (Fig 7D, lane 3). As anticipated, the GST-γb protein also failed to demonstrate helicase activity in reactions containing up to 9.7 pmol of GST-γb and ATP (Fig 7D, lanes 9–11). These results clearly demonstrate the helicase activity of the αa_HEL_ domain and the absence of γb helicase activity, and provided the basis to determine whether γb has an effect on αa helicase activity.

In order to test the ability of γb to enhance the MBP-αa_HEL_ helicase activity, we set up reactions containing ATP and a concentration of MBP-αa_HEL_ protein (4.4 pmol) that is below the threshold for release of detectable amounts of labeled 55 nt RNA from the 0.32 pmol dsRNA duplex. Control experiments in which up to 6.0 pmol of *E*. *coli* purified GST were added to the reaction failed to release substantial amounts of single-stranded RNA from the duplex substrate (Fig 7E, lanes 3–5, and 7F). However, when increasing amounts (1 to 6 pmol) of purified GST-γb were added to the assay mixtures, concentration dependent release of ssRNA was observed, culminating in up to 40% of the available single strand RNA (Fig 7E, lanes 6–8 and 12–14, and 7F). However, addition of the same molar concentrations of GST-γb_BM26_, which is deficient in ssRNA binding activity [34], failed to enhance release of single-stranded RNA (Fig 7E, lanes 9–11, and 7F). In conclusion, γb exhibits substantial enhancement of the αa RNA helicase function, and this activity requires the ability of γb to bind to ssRNA.

## Discussion

Viruses often remodel host intracellular membrane systems to establish specific subcellular microenvironments for replication. These events have been most extensively studied with BMV and TBSV virus replication complexes (VRCs), which induce host membrane remodeling and recruitment of virus replication proteins and gRNAs by replicase subunits to create morphologically distinct VRCs [49–52]. Our current studies new information verifying that the BSMV αa replicase protein promote γb targeting to the chloroplasts and that BSMV replicates in the chloroplasts. Moreover, our studies show that γb has a novel role in the enhancing helicase activities of the αa protein by functioning in helicase unwinding of dsRNA duplexes.

### Chloroplast localization of BSMV virus replication complexes

Early microscopy studies with BSMV revealed extensive chlorosis in leaves accompanied by pathological changes in chloroplasts, including cytoplasmic inclusions, membrane invaginations and distorted envelopes [35, 36, 53, 54]. Additional immunogold labeling studies revealed that dsRNAs are associated with invaginated chloroplast membranes and provided indirect evidence that BSMV establishes replication sites in these membranes [35, 37]. We also demonstrated that BSMV dsRNAs, BSMV plus- and minus-strand RNAs, and the BSMV replication proteins αa and γa, accumulate in association with chloroplasts (Fig 1).

Although γb is targeted to the chloroplast envelope and localizes to discrete chloroplast foci during BSMV infection, mechanisms underlying these observations were not explored [35]. Our sequence analyses with several online software programs (iPOSRT, Phobius, and ChloroP) failed to predict obvious chloroplast transit peptides within the γb protein, suggesting that other virus-encoded factors might be required to assist γb chloroplast targeting. As expected, protein-protein interaction assays verified interactions between the αa and γb proteins (Fig 3), and a γb mutant lacking the αa-interacting region is impaired in chloroplast associations (Fig 4E). These results therefore provide a persuasive argument that chloroplasts have a central role in the replication of BSMV, and that αa and γb protein interactions are critical for establishing chloroplast associations.

We also observed that a portion of the fluorescence of a γb-GFP fusion protein accumulates the cytoplasm and that γb fluorescent puncta accumulate at the periphery of the cell (Figs 3A and 4E). It is of particular interest that TGB2 also co-localizes with the chloroplasts in association with γb [35]. The fact that TGB2 participates in formation of talin intermediates and membrane proliferation associated with BSMV infection [55] and is localized to the cell wall by TGB3 [56] evokes potential mechanisms whereby γb-TGB2 interactions can mediate γb trafficking from chloroplasts to the cell periphery. This hypothesis also raises the possibility that γb may have additional activities involving subcellular trafficking and may participate in cell-to-cell transit.

### Efficient replication of BSMV requires novel γb functions

The phenotypes resulting from γb-deficient BSMV infections were previously thought to be primary a consequence of γb RNA silencing suppressor activities [31]. However, when the start codon of γb ORF was mutated in RNAγ to eliminate γb protein expression during infection, we observed dramatically reduced gRNAα accumulation relevant to accumulation of plus-strand α and γ RNAs (Figs 5B and 6C), as was also noted in previous experiments in barley protoplasts infected by BSMV γb deletion mutants [26]. Our current results extend these early findings by revealing that disruption of γb silencing suppressor activity has only moderate effects on accumulation of plus-strand BSMV gRNAα (Fig 5B). Moreover, TBSV p19 and potyvirus HCPro VSR proteins expressed in transgenic *N*. *benthamiana* failed to restore RNAα accumulation of Δγb RNA mutant infections to the levels of wild-type BSMV infections (Fig 6 and S11 Fig). In addition, ectopic γb expression from RNAβ or from transgenic *N*. *benthamiana* only partially restored the replication of γb-deficient BSMV (Fig 6 and S10 Fig). We hypothesize that this phenomenon may be a consequence of abnormal kinetics or levels of ectopic γb expression, and that sophisticated regulation of γb expression from sgRNAγ is required for fully effective functions during infection.

BSMV γb protein expression only occurs after the initial stages of replication have commenced. Although, it has not been technically possible to dissect very early events in synchronous infections, substantial periods may be devoted to minus-strand RNA synthesis from the gRNA templates after translation of the replicase subunits. Subsequently, plus-strand gRNA synthesis and possibly specific regulation of the timing and translation of sgRNAs may occur depending on γb effects on individual gRNAs. This may be related to effects of γb expression on minus-strand RNAα accumulation.

It is possible that the γb protein may have as yet unrecognized roles in accurate translation of RNAα to produce functional αa proteins. This suggestion relates to findings by Zhou *et al*. [27] showing that BSMV RNAα mutants that are unable to translate functional αa protein from RNAα fail to replicate in protoplasts after co-transfection with wt RNAs α and γ. In contrast, replication of RNAβ and RNAγ deletions was not affected substantially, and in some cases, RNAγ deletions exhibited such high levels of replication that they exhibited properties of defective interfering RNAs [27]. *Cis*-preferential replication is a conserved viral replication strategy used by many plant and animal RNA viruses to efficiently recognize and select against nonfunctional gRNAs, and selectively replicate only functional gRNAs [57–64]. In one notable example of *cis*-preferential replication, *Brome mosaic virus* (BMV) RNA1 replication requires translation of a functional 1a replicase protein, and expression of the 1a protein *in trans* is unable to support defective RNA1 replication [59]. *Tobacco mosaic virus* (TMV) replication proteins also exhibits similar *cis*-translation effects in which functional 126 and 183 kDa replicase proteins must be translated to ensure *cis*-replication of the gRNA [60]. In this case, *cis*-preferential replication requires co-translational binding of the 126 kDa replicase protein to the translated gRNA [61]. Similarly, BSMV replication of RNAα depends on *cis*-translation of the αa protein [27] and it is possible that γb binding to the αa protein may directly enhance the *cis*-preferential replication of RNAα through enhancing αa helicase activity.

### Helicases encoded by either hosts or viruses do not act alone

Recent studies indicate that the *Arabidopsis thaliana* AtRH8 and AtRH9 DEAD-box RNA helicases are essential host factors required for potyvirus infections [17, 65]. The AtRH8 and AtRH9 helicases interact with *Plum pox virus* VPg and *Turnip mosaic virus* NIb (RdRp) proteins, respectively, and are recruited to VRCs during potyvirus infections [17, 65]. Similarly, plant DEAD-box RNA helicases AtRH2 and AtRH5 are co-opted by TBSV to promote asymmetric viral RNA replication by coordinating release of replicating RNAs from the template RNAs. Other host helicases, AtRH20 and AtRH2, work coordinately to maintain TBSV genome integrity and suppress viral recombination [14, 16]. Furthermore, recent studies of the mechanisms underlying TBSV replication reveal that a host DEAD-box RNA helicase directly enhances viral plus-strand RNA synthesis [13]. DEAD-box helicases were also found to participate in the replication of many animal RNA viruses [8]. Despite the indispensable roles of RNA helicases in the plus-strand virus infections described above, the factors involved in regulating RNA helicases during the progression of the viral replication remain largely unknown. Our study reveals a novel role for the γb VSR protein by identifying functions in enhancing unwinding of RNA duplexes during viral replication. Nevertheless, highly conserved sequences across diverse cysteine-rich VSR proteins [See Fig 10 in Reference 19] implies that enhancement of replicase helicase activities during interactions with γb and related VSR proteins may not be restricted to the hordeiviruses. Also, sequences across diverse RNA virus helicases are highly conserved (S1 Fig), and various host RNA binding proteins were utilized in RNA virus replication [66], the helicase enhancer role for γb described in our work provides important implications for the existence of non-helicase factors modulating the helicase activity in the replication of plus-strand RNA viruses.

### Model for γb interactions during early stages of infection

**Fig 8:**
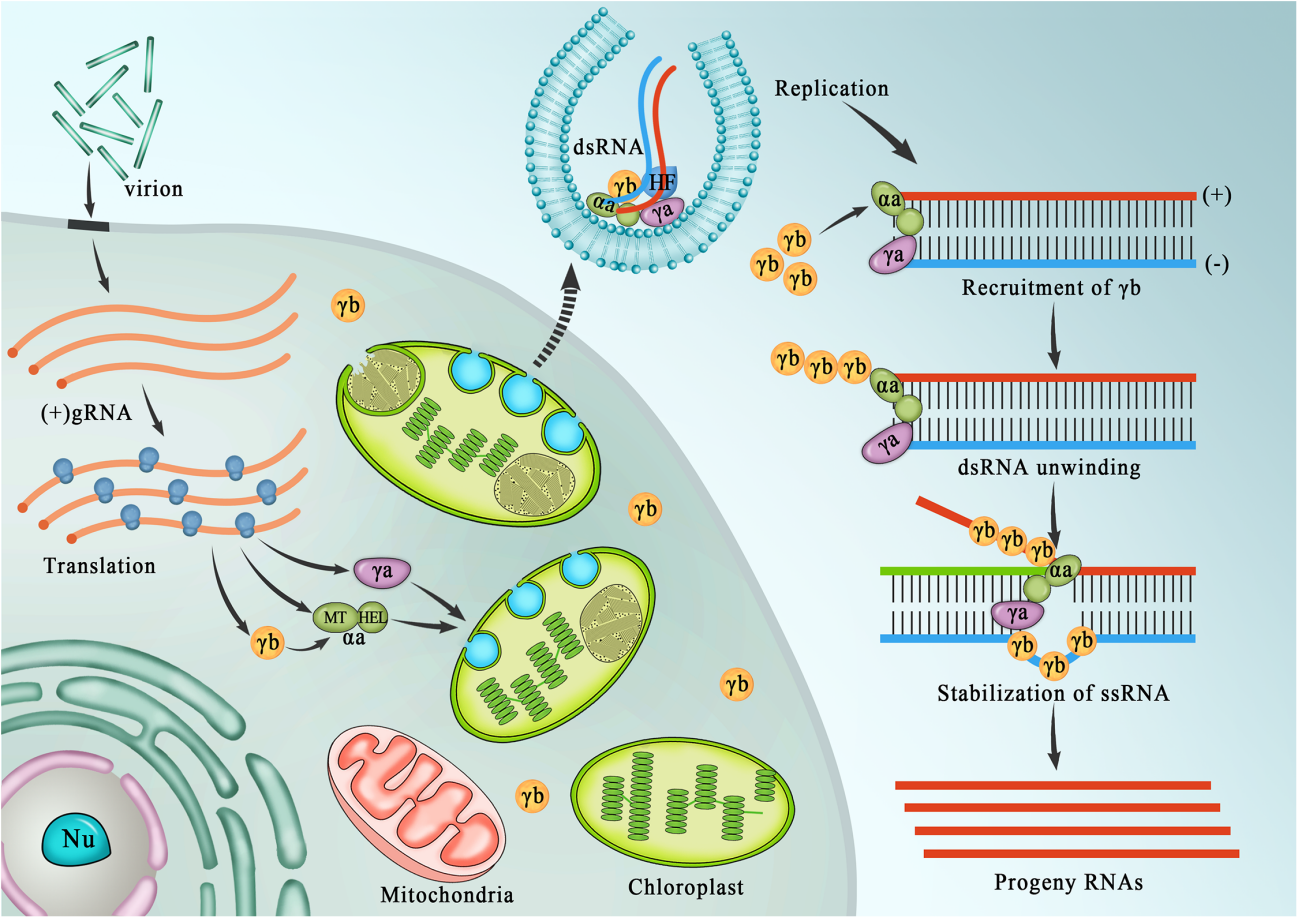
Model outlining the roles of γb in BSMV replication. Model emphasizing γb activities during the BSMV RNA replication cycle. During initial stages of replication, the αa and γa subunits are translated from the genomic (g) RNAs and form heterologous associations with putative host factors (HF) needed for assembly of the viral replication complex (VRC). The αa replicase subunit interacts with the γa replicase, and facilitates chloroplast localization. After assembly of replication competent VRCs, the viral replicase uses the parental gRNA as a template for synthesis of minus-strand gRNA, and the two strands interact closely to form a dsRNA replication template. During early stages of replication, the dsRNA may function as a template for synthesis of plus-strand progeny RNAs that initially function in translation of the αa and γa replicase subunits. Subsequently, synthesis of sgRNAs is initiated from the minus-strand template, and the γb proteins are translated and recruited to chloroplasts by αa. The γb proteins then bind to the αa subunit where enhance αa helicase activities to stimulate asymmetric synthesis of progeny plus-strand RNAs from the minus-strand templates.

Based on our cumulative studies of BSMV, we propose a general model to describe the initial stages of hordeivirus replication and the novel roles of γb and αa-γb interactions in these processes (Fig 8). During the initial stages of replication, gRNAs are released from virions, the αa and γa subunits are translated and form heterologous associations with host components needed for replicase functions. The αa subunits then interact with γa and host proteins to form replicase complexes that are transported to chloroplasts where they initiate membrane modifications needed for formation of nascent VRCs. The RdRp initiates synthesis of minus-strand gRNAs that begin to accumulate in the VRCs. As infection proceeds, a switch to plus-strand gRNAs and sgRNA transcription commences, and γb and the triple gene block proteins are translated. The newly synthesized γb protein binds to the αa protein and is transported to the chloroplasts where it functions to enhance αa helicase activities during copying of minus-strand templates to stimulate increased levels and stability of plus-strand gRNAα. This process, which is essential for elevated RNAα synthesis, eventually results in accumulation of large amounts of plus-strand progeny gRNAs compared to minus-strand gRNA replication templates. The γb protein subsequently serves to suppress interactions of host silencing complexes that facilitate stability of the progeny gRNAs, and may interact with the TGB proteins during subsequent cytoplasmic and cell-to-cell movement events.

In conclusion, our study provides a more sophisticated understanding of the multifunctional roles of VSR proteins in the regulation of plus-strand RNA virus replication and provides a basis for additional studies to explore mechanisms whereby VSR protein and other host factors function during replication of plus-strand RNA viruses.

## Materials and methods

### Plant growth conditions

*N*. *benthamiana* plants were grown in a climate controlled chamber with a 16 h light (~75 mmol/m^2^. s) and 8 h dark photoperiod at 23–25°C as described previously [67].

### Plasmid constructions

For transient expression, various γb-derivatives were amplified from the RNAγ cDNA and then cloned into the binary vectors pGD [43] or its derivative pGDRm to generate the plasmids shown in S2B Fig. For subcellular localization of the viral replicase proteins, the αa and γa ORFs were cloned into pSuper1300-GFP, [68]. For co-immunoprecipitation experiments, the *gfp* gene or 3×Flag-tagged γb was cloned into the pDONR/Zeo entry plasmid (Invitrogen) by Gateway LR recombination, and then transferred into pGWB14 destination vectors [69] by recombination to produce plasmids for expression of GFP-HA and γb-3×Flag. The pGWB5-γb-GFP plasmid was constructed by engineering the γb gene into the pGWB5 vector [69] using the Gateway cloning system (Invitrogen).

Several plasmids used in this study, such as the pCaBS-α, pCaBS-β, pCaBS-γ, and pCa-γbLIC, were constructed by Yuan *et al*. [70]. RNAγ cDNA containing the GFP sequence in-frame with γb was amplified from the pT7-γ/γb:GFP plasmid [71], and then cloned into the pCass4-Rz binary vector [72] to generate the pCaBS-γ_γb-GFP_. In addition, the fragment encompassing the *Bgl* II and *Bam*H I restriction sites of RNAγ cDNA was amplified and cloned into pUC18 to generate the intermediate pUC18-A plasmid. All subsequent molecular engineering of the γb ORF, including deletions, point mutations, substitutions, and fluorescent protein fusions were generated from the pUC18-A plasmid, followed by digestion with specific restriction enzymes and ligation into pCaBS-γ, pCa-γbLIC, or pCaBS-γ_γb-GFP_ to obtain the plasmids shown in S2C Fig. For viral RNA visualization, specific RNA sequences recognized by half YFP-fluorescent fused PUMHD polypeptides [38] were inserted downstream the termination codon of γb in plus or minus sequence orientations by reverse-PCR, followed by fragment transfers to the pCaBS-γ plasmid using ligation-independent cloning with the Seamless Assembly Cloning Kit from Clone Smarter (S2C Fig)

For yeast two-hybrid (Y2H) and BiFC assays, various αa and γb-derivatives were amplified from pCaBS-α and pCaBS-γ. The resulting fragments were recovered and engineered into either the GAL4-based Y2H assay pGBKT7 and pGADT7-Rec (Clontech) vectors, or into the pSPYNE-35S and pSPYCE-35S split YFP destination vectors [73] by standard procedures [74] or with the Seamless Assembly Cloning Kit from Clone Smarter.

For protein purification, an empty vector pGEX-KG [75] was used for to facilitate expression of GST proteins. Genes encoding the γb and γb_BM26_ derivatives (^25^RK^26^→^25^QN^26^) were cloned into the pGEX-2T vector (GE Healthcare) using standard procedures [74]. To generate pDB.His.MBP-αa_HEL_, amino acids 614–1138 of the αa protein was cloned into the plasmid pDB.His.MBP, which was obtained from DNASU Plasmid Repository (https://dnasu.org/DNASU/Home.do).

The primers used for constructing these plasmids are listed in S1 Table and sequence analyses were performed to confirm the accuracy of all the plasmids.

### Agroinfiltration and GFP imaging of suppressor activities

*Agrobacterium tumefaciens* EHA105 was transformed with plasmids harboring 35S-driven expression cassettes by a freeze-thaw method as previously described [76]. *A*. *tumefaciens* was cultured in LB medium (containing 100 mg/L Kan and 25 mg/L Rif) for 10–16 h at 28°C by shaking at 220 rpm. *Agrobacterium* cells were then collected by centrifugation at 3000 *g* for 10 min, followed by resuspension in infiltration buffer (10 mM MgCl_2_, 100 μM acetosyringone, and 10 mM MES, pH 5.8), and incubated at 28°C for at least for 2 h prior to infiltration.

In order to evaluate the suppression activity of various γb derivatives on RNA silencing, different combinations of equal amount of bacterial suspensions with optical density at 600 nm (OD_600_) value of 0.5, harboring 35S-GFP or plasmids expressing various γb derivatives, were co-infiltrated into abaxial leaves of 4–5 week-old *N*. *benthamiana* with a needle-free syringe [40, 41]. At 3 dpi, *N*. *benthamiana* plants were illuminated with a long-wave ultraviolet lamp (B-100AP/R, UVP) for observations of suppressor activity, and photographed using a digital camera (CoolPix 4500, Nikon) with a yellow filter (Gelatin filter No. 15, Kodak). Similar procedures were carried out for agroinfiltration of other BSMV derivatives.

### Generation of γb transgenic *N*. *benthamiana* plants

The pGWB14-γb-3×Flag plasmid was introduced into *Agrobacterium* strain EHA105, followed by leaf disc transformation of *N*. *benthamiana* plants as described previously [77]. After cultivation and regeneration of leaf explants, genomic DNA was isolated with a standard CTAB method [78], and PCR analysis was conducted to screen the positive transgenic plants (S1 Table and S9 Fig).

### Isolation of *N*. *benthamiana* chloroplasts

Isolation of *N*. *benthamiana* chloroplasts was performed by a minor modification of a previous report [79]. Briefly, *N*. *benthamiana* was placed in a dark chamber for 10 h to reduce the starch content of the chloroplasts before harvesting leaf tissues. Next, about 2 g of tissue from agroinfiltrated 4-week-old *N*. *benthamiana* plants were collected. After removing the primary veins and petioles, the leaf blades were ground in a mortar and pestle with 6 ml of pre-cooled buffer (300 mM sorbitol, 0.5 mM MgCl_2_, 1 mM EDTA, 50 mM HEPES-KOH, 1 mM DTT, and 0.1% BSA, pH 6.8) [80]. Tissue brei was removed by filtration through Miracloth (Sigma-Aldrich), and the pellet resulting after centrifugation at 1000 *g* for 10 min was re-suspended in 1 ml of suspension buffer (0.3 M sorbitol, 0.5 mM MgCl_2_, 50 mM HEPES-KOH, pH 8.0, 10 mM K_2_HPO4, 1 mM DTT, and 0.1% BSA). The chloroplasts were then enriched over a Percoll step gradient (40% and 80% Percoll in suspension buffer), centrifuged at 13000 *g* for 15 min., and the intact chloroplasts concentrating at the interface between the Percoll solutions were recovered with a pipette and washed by centrifugation to remove residual Percoll and BSA before confocal microscopic analysis. All operations above were conducted at 4°C or on the ice.

### Confocal laser scanning microscopy

Agro-infiltrated *N*. *benthamiana* leaf tissues were observed with a Zeiss LSM-710 confocal microscope equipped with Zeiss Zen 2012 software. GFP, YFP and RFP fluorescence, and chlorophyll auto-fluorescence was visualized under 488 nm, 514 nm, 543 nm and 633 nm respectively with an argon laser. Images were captured digitally with a Zeiss Axiocam camera and processed with Imaris 7.4.2 software (Bitplane). To avoid crossfluorescence effects between neighboring GFP and YFP emission spectra, a sequential scanning mode was used for image capture at a 1024 by 1024 pixel resolution.

### Analysis of viral RNAs

Northern blot analyses were performed as described previously with minor modifications [81, 82]. Briefly, leaf samples from agro-infiltrated *N*. *benthamiana* plants were harvested and total RNA was extracted [83]. The RNA was quantified using a NanoDrop ND-1000 (Thermo Fisher Scientific) and denatured at 68°C for 10 minutes in a buffer consisting of 1:5 and 1.8 V/V ratios of formamide and formaldehyde in 10×MOPS buffer (200 mM MOPS, 50 mM NaOAc, 10 mM EDTA, pH 7.0). For the detection of BSMV plus-strand RNA, 5 μg of total RNA was separated over 1.2% agarose/1.1% formaldehyde gels, and 30 μg total RNA was used to detect BSMV minus-strand RNA accumulation. The RNA was vacuum-blotted onto Hybond-N^+^ nylon membranes (GE Healthcare), fixed by UV crosslinking and stained with methylene blue solution (0.04% methylene blue, 500 mM NaOAc). The 294 nt 3′-UTR and 1 kb RNAα-specific cDNA fragments were cloned into pSPT18 and pSPT19 (Roche), respectively, which were linearized for *in vitro* transcription of plus- and minus-strand RNA probes (S1 Table). After overnight hybridization at 65°C and post hybridization washes, the nylon membranes were exposed to a Storage Phosphor Screen (GE Healthcare) for 48–72 h, and the resulting data was digitized with a Typhoon 9400 PhosphorImager (GE Healthcare) using ImageQuant software. For plus-strand RNA detection, some blots were stripped by boiling in l×SSC, 0.1% SDS for 10 min and reprobed with [α-^32^P]UTP-labeled minus-strand RNA *in vitro* transcripts.

### Statistical analysis

The intensity of the RNAα-specific bands as well as methylene blue stained 18S rRNA loading controls were quantified by Quantity One software (Bio-Rad), respectively, and quantitative value derived from various RNAα-specific bands were then normalized to their 18S rRNA loading controls followed by calculation with respect to the value of RNAα from that of pCaBS-α + pCaBS-γ_γb_ (i.e. wtRNAα + wtRNAγ) -infiltrated leaves in each panel, which was set to 100%. Quantified data in each panel were then subjected to statistical analysis using SPSS software (version 22.0, IBM). The data were compared using one-way analysis of variance (ANOVA). Significant differences in the RNAα accumulation were determined by Duncan's multiple range test.

### Expression and purification of recombinant proteins

All plasmids used for protein expression were transformed into *E*. *coli* strain BL21 (DE3) pLysS cells (Novagen) by standard procedures [74]. *E*. *coli* cells were cultured at 37°C for about 4 h, followed by addition of 200 μM isopropyl β-D-1-thiogalactopyranoside (IPTG, Sigma-Aldrich) and shaking at 18°C for 18 h for induction of protein expression. Purification of recombinant proteins was performed as described previously [67, 84]. Briefly, *E*. *coli* cells were harvested, re-suspended in buffer T (20 mM Tris-HCl, pH 7.5, 500 mM NaCl, 10% glycerol, 1 mM PMSF), disrupted by ultrasonication (Model 500 Homogenizer, Fisher Scientific), and centrifuged to obtain the clarified supernatant. For purification of the MBP-tagged αa_HEL_ protein, supernatants were passed over Ni−NTA agarose columns (Bio-Rad) at least three times to ensure efficient binding to the beads, and the columns were eluted using stepwise increases in imidazole concentration. For recovery of GST-tagged proteins, supernatants recovered after centrifugation were passed over a glutathione-Sepharose affinity column (GE Healthcare), and the GST fusion proteins were eluted with T buffer containing 60 mM L-Glutathione and 2 mM DTT. The eluted recombinant proteins were concentrated with an Amicon Ultra-15 filter unit (Millipore) and used for RNA helicase assays.

### Analysis of protein expression

Agroinfiltrated *N*. *benthamiana* leaves were harvested and homogenized in liquid nitrogen, followed by addition of equal volumes of gel loading buffer (100 mM Tris-HCl, pH 6.8, 20% glycerol, 4% SDS, 200 mM β-mercaptoethanol, 0.2% bromophenol blue). After vortex mixing and boiling, the samples were centrifuged for 10 min at 12000 *g*. Proteins remaining in the supernatant were resolved by 12.5% SDS-PAGE, followed by staining with Coomassie brilliant blue or were transferred to nitrocellulose filters for Western blot analysis [85].

Western blots were performed as described previously [67, 84]. Briefly, nitrocellulose membranes containing transferred proteins (Hybond-C, GE Healthcare) were blocked, incubated with primary antibodies raised against the γb, Actin, GFP, FLAG or HIS proteins. After washing, the membranes were incubated with secondary antibodies conjugated to horseradish peroxidase or protein A-alkaline phosphatase (Sigma-Aldrich), and the signals were detected with an enhanced chemiluminescence (ECL) detection kit (GE Healthcare) or by color reactions developed by incubating with substrate solution (0.33 mg/mL NBT and 0.165 mg/mL BCIP in 100 mM Tris-HCl buffer, pH 9.5, containing 100 mM NaCl). The results were recorded with a Cannon scanner.

### Yeast two-hybrid assays

Yeast two-hybrid (Y2H) assays were performed using the GAL4 system as described previously [86]. Various γb and αa derivatives were cloned into the pGADT7-Rec or pGBKT7 vectors (Clontech), followed by transformation into the AH109 or Y187 yeast strains by a lithium acetate method [87]. Colony-PCRs were conducted to identify positive transformants, and the transformed yeasts AH109 (a mating type) were mated with yeast Y187 (α mating type) in a 5 mL tube containing 500 μL of 2×YPDA culture. After 20, cells were plated onto SD/-Leu/-Trp or SD/-Ade/-His/-Leu/-Trp synthetic drop-out media supplemented with 10 mM 3-amino-1,2,4-triazole (Life Technologies), and cultured at 30°C for 5 days. Interactions between different proteins were assessed by observing yeast cell growth and by the appearance of lacZ blue color after addition of α-X-Gal (Sigma-Aldrich) substrate.

### Coimmunoprecipitation (co-IP) assays

Co-IP experiments were performed by slight modifications of a previously described method [88]. *N*. *benthamiana* leaves agroinfiltrated with different expression vectors were pooled and ground in liquid nitrogen as described above. Leaf powders were transferred to a 50 mL centrifuge tube and mixed with 3 volumes (w/v) of GTEN buffer [10% glycerol, 50 mM Tris-HCl, pH 7.5, 1 mM EDTA, 150 mM NaCl, 10 mM DTT, 2% (w/v) polyvinylpolypyrrolidone (PVPP), 1% protease inhibitor cocktail (Sigma-Aldrich), 1% Triton X-100, 0.15% (v/v) NP-40], mixed by vortexing, and incubated in an ice bath for 30 minutes, followed by centrifugation at 1000 *g* for 20 min. The resultant supernatants were diluted in 2 volumes (w/v) of modified GTEN buffer without detergent and PVPP to obtain the appropriate concentrations for subsequent affinity chromatography. The mixtures were then incubated overnight at 4°C on a rocker platform with anti-FLAG M2 affinity gel (Sigma-Aldrich), followed by washing with a modified GTEN buffer. Western blots were performed to analyze the IP products by using antibodies against different proteins.

### RNA helicase assays

Partial double-stranded RNA (dsRNA) substrates were prepared [89, 90]. In a separate reaction, the *BsrG* I-digested pSPT19 (Roche) plasmid was transcribed with T7 RNA polymerase to produce a 149 nt template strand, followed by DNase I treatment, phenol-chloroform extraction, and ethanol precipitation. A chemically synthesized 55 nt RNA release strand was purchased from Takara, followed by 5′-end labeling with [γ-^32^P]ATP using T4 polynucleotide kinase. The labeled 55 nt RNAs were then annealed to the 149 nt RNA transcription products by mixing an approximately 1:3 molar ratio of the two RNA strands in hybridization buffer (500 mM NaCl, 25 mM HEPES-HCl, pH 7.4, 1 mM EDTA, 0.1% SDS), and heating the mixture at 95°C for 5 min, 55°C for 30 min, followed by overnight incubation on a shaker at low speed. Hybridization reactions were ethanol precipitated and re-suspended in TE buffer (10 mM Tris-HCl, pH 7.5, 1 mM EDTA). The annealed products contained 90 nt 3′ and 4 nt 5′ overhanging regions on the 149 nt strand, and an internal double-stranded region consisting of the 55 nt RNA release strand (Fig 7C and S2 Table).

RNA unwinding assays were carried out as previously described [89, 91] by incubating 4.4 pmol MBP-αa_HEL_ helicase protein with 0.32 pmol purified partial dsRNA substrate at 37°C for 2 h in dsRNA unwinding buffer (25 mM MOPS-KOH, pH 6.5, 5 mM ATP, 3 mM MnCl_2_, 2 mM DTT, 2 U/μL RNasin). Reactions were terminated by adding 5 μL of 5×RNA sample buffer (100 mM Tris-HCl, pH 7.5, 50 mM EDTA, 0.1% Triton X-100, 0.5% SDS, 50% glycerol, 0.1% bromophenol blue) and electrophoresed on 15% native polyacrylamide (Acr/Bis = 37.5:1) gels in 0.5×TBE buffer at room temperature until the bromophenol blue dye approached the bottom of the gels. The gels were dried under vacuum in a Bio-Rad 583 gel dryer and exposed to a Storage Phosphor Screen (GE Healthcare). Single-stranded and duplex RNA bands were visualized with a Typhoon 9400 PhosphorImager (GE Healthcare) and quantified using Quantity One software (Bio-Rad).

## Supporting information

S1 Table Primers used for vector construction in this work. (PDF)

S2 Table Sequences of single-stranded RNA used for preparation of the partial dsRNA duplex. Underlined letters constitute the complementary regions of the partial dsRNA duplex.
(PDF)

S1 Fig Sequence comparisons of the BSMV αa and TGB1 helicase domains with helicase domains of other RNA virus proteins. Common or related amino acid residues are shown in red text and highlighted in yellow. “Xn” indicates the number of variant amino acids between the conserved domains.
(TIF)

S2 Fig Schematic depiction of plasmids used in this study. **Panel A**: BSMV Genome organization with functional regions of the γb protein highlighted. **Panel B:** Illustration of γb plasmids used for transient expression of γb and various γb derivatives in *N*. *benthamiana* leaf tissues. **Panel C**: Diagram of various pCaBS plasmids harbored in *A*. *tumefaciens* for delivery of infectious BSMV RNA derivatives after agroinfiltration of leaf tissues. RNAs transcribed in cells after agroinfiltration of the pCaBS-α, pCaBS-β, and pCaBS-γ or pCaBS-γ_Δγb-GFP_ vectors were sometimes designated BSMV RNAs α, β, γ, γ_Δγb-GFP,_ etc. **Note:** The γa replicase was inactivated by mutating the GDD motif to GAD.
(TIF)

S3 Fig Additional evidence for chloroplast localization of plus-strand and double-stranded BSMV RNAs in systemically infected *N*. *benthamiana* leaves. **Panel A:** Leaves were agroinfiltrated with RNAα + RNAβ + RNAγ_(+)γbPUM_ as in Fig 1. The white arrow indicates the nucleus (N). Arrowheads indicate the cytoplasm-localized plus-strand BSMV RNAs. Scale bar, 10 μm. **Panel B:** Symptomatic leaves of BSMV-infected *N*. *benthamiana* plants were co-infiltrated with the split YFP-tagged FHV B2 and Marburg virus VP35 proteins. Images are the overlay of GFP channel and chlorophyll autofluorescence (Chl). Scale bar, 10 μm. Note: The figure organization and relevant figure designations can be found in the Fig 1 legend.
(TIF)

S4 Fig Additional evidence supporting the suppressor activities of γb C-terminal fluorescent protein fusions. **Panel A**: *N*. *benthamiana* leaves were agroinfiltrated with *A*. *tumefaciens* harboring plasmids (see S2B Fig) for expression of γb (positive control), γb-GFP or γb-RFP for the spot silencing assay, and then photographed under long wavelength UV illumination at 3 dpi. (EV = empty pGD vector for use as a negative control). **Panel B**: Western blot analysis of the expressed proteins in agroinfiltrated leaves. Bands corresponding to the reporter proteins were shown on the left (arrowheads), and antibodies used for protein detection are shown on the right side of each blot. Equal protein loading is suggested by the similar amounts of actin protein (bottom panel).
(TIF)

S5 Fig BiFC assays showing interactions between the αa and γb proteins of *Lychnis ringspot virus* (LRSV) and *Poa semilatent virus* (PSLV). **Panels A and B**: BiFC assays to detect interactions between the αa and γb proteins of LRSV and PSLV. The experimental design was described in the legend to Fig 3A, and confocal microscopic analyses were carried out at 3 dpi. Various constructs used for agroinfiltration are indicated on top left corner of each panel, while the channels used for imaging are shown on the left. The YFP signal is false-colored green. DIC, Differential interference contrast. Chl, chlorophyll autofluorescence (in red). Scale bar, 10 μm. **Note**: The LRSV and PSLV αa and γb binding and self-interactions are similar to those of BSMV. However, the fluorescence locations of both the LRSV and PSLV positive αa-γb interactions appear to be distinct from chloroplast autofluorescence, suggesting that these viruses and BSMV may replicate in different subcellular sites.
(TIF)

S6 Fig Western blot analysis of YFP proteins from leaf tissues agroinfiltrated with *Agrobacteria* containing BiFC constructs. Detection of half-YFP fusion proteins in agroinfiltrated leaves at 3 dpi. Various combinations of the -cYFP and -nYFP derivatives shown at the top of the panel correspond to those shown in Fig 3A. Sizes (in kDa) of molecular weight markers (Mr) are shown on the left and the antibodies used for detection are shown on the right. Arrowheads indicate the target proteins. H, Healthy leaf control. Equal protein loading was assessed by Actin detection (bottom panel).
(TIF)

S7 Fig Analysis of GFP expression in leaves co-infiltrated with *Agrobacteria* for expression of RNAα and RNAγ variants. **Panel A**: Schematic representation of RNAγ GFP constructs. **Panel B**: Diagram of the leaves co-infiltrated with RNAα and the RNAγ GFP variants illustrated in S7A Fig. **Panel C**: Western blot analysis of total protein samples from leaf tissues co-infiltrated with RNAα and RNAγ-derived constructs (OD_600_ = 0.1) using antibodies against GFP. Equal protein loading was assessed by Actin detection (bottom panel). H, healthy leaf control. **Note**: Small bands (asterisks) appearing below the γb-GFP fusion variants (arrowheads) in lanes 2 and 4 probably were due to free GFP expression mediated by recombination of RNAγ_γbGFP_ variants. Equal protein loading was assessed by Actin detection (bottom panel). **Panel D**: Confocal microscopic analysis of leaf tissues co-infiltrated with RNAα and RNAγ-derived constructs. The concentrations of *Agrobacterium* cells used for agroinfiltration are shown on the left sides of the horizontal images. Scale bar, 100 μm.
(TIF)

S8 Fig BSMV γb provides a pathogenesis determinant that affect symptom production during infection of *N*. *benthamiana* and barley. **Panel A**: Symptom phenotypes of *N*. *benthamiana* plants co-infiltrated with *Agrobacteria* expressing RNAα, RNAβ, and various RNAγ-derivatives shown above the upper image. The lower image shows representative symptoms of upper uninoculated leaves corresponding to the plants shown above. The numbers beneath the lower panel indicate the numbers of plants expressing systemic symptoms among 12 inoculated *N*. *benthamiana* plants at 24 dpi. **Note:** Only those plants inoculated with wtBSMV exhibit visible symptoms. **Panel B**: Western blot analysis of protein samples from upper uninoculated leaves shown in the lower panel of S8A Fig. Antibodies used for detection are indicated on the right. Equal protein loading was assessed by Actin detection (bottom panel). **Panel C**: Systemic symptoms of barley leaves mechanically co-inoculated with *in vitro* transcripts of RNAα, RNAβ, and various RNAγ-derivatives shown above each image. The numbers beneath the lower images indicate the numbers of barley plants that display systemic symptoms among 12 inoculated plants at 54 dpi. **Panel D**: Western blot analysis of the total protein samples from upper uninoculated leaves of the barley leaves as shown in S8C Fig. Antibodies used for detection are indicted on the left. Equal protein loading was assessed by Actin detection (bottom panel). Arrowheads between the two horizontal panels indicate the target band of corresponding viral protein. **Note**: a band that we suspect to be a γb doublet (asterisk) is present in panels B and D. In addition, the γb mutant derivatives result in very low levels of expression of the CP and TGB1 as has frequently been observed previously.
(TIF)

S9 Fig **Phenotypic observations (Panel A) and PCR amplification (Panel B) of p19- and γb-transgenes from *N*. *benthamiana* plants**. NT, non-transgenic. The asterisk indicates the non-specific band.
(TIF)

S10 Fig Expression of γb protein either *in trans* or under the control of the RNAβ TGB1 subgenomic RNA promoter failed to restore RNAα replication of γb-deficient BSMV. **Panel A**: Schematic representation of plasmids used for agroinfiltration of *N*. *benthamiana*. **Panel B**: Molecular analysis of the replication of γb-deficient BSMV in the agroinfiltrated *N*. *benthamiana* leaves by Western blot (I and II), and Northern blot (III-V). Lanes 1–2, negative and positive control leaves agroinfiltrated with *Agrobacteria* strains for co-expression of wild-type RNAα + RNAγ_γaGDDm_, and wild-type RNAα + wild-type RNAγ. Lane 3, leaves co-expressing RNAα, γb-deficient RNAγ (Δγb) or γb_ATGm_), and a modified RNAβ (CP_ATGm_ΔTGB1) mutant unable to express TGB1, TGB2, and CP. Lane 4, co-expressions identical to Lane 3 except that γb_ATGm_ was included in the infiltration mixture instead of the Δγb mutant. Lane 5, leaves co-infiltrated for expression of RNAα, a γb-deficient mutant RNAγ (Δγb) and a modified RNAβ with γb substituted for the TGB1 ORF (CP_ATGm_ΔTGB1-γb shown in S10A Fig). Lane 6, identical to lane 5 except that the infiltration mixture included γb_ATGm_ instead of Δγb. Lanes 7 and 8, leaves co-infiltrated for expression of RNAα, and γb and the γb-deficient RNAγ mutants Δγb or γb_ATGm_, respectively. For Western blot analysis, the γb- and Actin-specific antibodies for protein identification are shown on the right. Equal protein loading was evaluated by Actin detection (panel II). For Northern blot analysis, the probes used for detection of the plus-strand (+) RNAs are shown on the left. Bands corresponding to RNAα, RNAβ, RNAγ, and subgenomic RNAγ (see *-labeled bands, γ_sg_), or subgenomic RNAβ (see arrowhead-labeled bands, β_sg_) are indicated along the right side of each panel. Methylene blue staining of rRNAs was used as a loading control (panel V). The experiments were independently repeated twice and similar results were obtained.
(TIF)

S11 Fig Inability of *trans* expression of the HCPro VSR protein to restore RNAα accumulation in BSMV γb-deficient infected leaves. **Panel A**: Gene silencing suppression abilities of HCPro-transgenic *N*. *benthamiana*. Non-transgenic (NT) and HCPro-transgenic plants were co-infiltrated with 35S-GFP plus the empty pGD vector (EV) and observed at 3 dpi under UV illumination. Transient coexpression of 35S-GFP and HCPro, and 35S-GFP and γb in non-transgenic plants served as positive controls. **Panel B**: Western blot analysis of total protein samples from agroinfiltrated *N*. *benthamiana* leaves using a GFP-specific antibody. Equal protein loading was assessed by Actin detection. **Panel C**: Northern blot analysis of plus-strand BSMV RNAα (Top blot) or BSMV α, and γ plus-strand RNAs (Bottom blot). *N*. *benthamiana* leaves were agroinfiltrated with *Agrobacteria* strains for expression of RNAα and the RNAγ-derivatives shown above the panel lanes. Bands indicative of RNAα, RNAγ, and sgRNAγ (γ_sg_) are indicated on the right. Methylene blue staining of rRNAs was used as a loading control. Blots are representative of two independent experiments with similar results.
(TIF)
