# Supplementary material for: The Barley stripe mosaic virus γb protein promotes chloroplast-targeted replication by enhancing unwinding of RNA duplexes
Source: PLoS Pathog. 2017 Apr 7;13(4):e1006319. doi: 10.1371/journal.ppat.1006319 (PMC5397070; doi:10.1371/journal.ppat.1006319)
Supplement: S1 Table — (PDF) [file ppat.1006319.s001.pdf]

S1 Table. Primers used for vector construction in this work

Primer sequence (5'-3') [a]		Position and description [b]	Purpose	Experiment
F_{DONR}-γb	GgggacaagtttgtacaaaaaagcaggctATGATGGCTACTTTCTCTTG	RNAγ nt 2081-2100	pDONR-γb<br>pGWB5-γb (γb-GFP)<br>pGWB14-γb-3×Flag	Subcellular localization<br><br>Co-IP<br><br>PCR analysis of γb transgenic plant
R_{DONR}-γb	GgggaccactttgtacaagaaagctgggtcCAACTTAGAAACGGAAGAA	RNAγ nt 2537-2518		
R_{DONR}-γb3Flag	GgggaccactttgtacaagaaagctgggtcAAGCTTGTCATCGTCATCCTTG	3Flag nb 81-60		
F_{DONR}-GFP	GgggacaagtttgtacaaaaaagcaggctATGAGCAGTAAAGGAGAAGAACT	eGFP nb 1-23	pGWB14-GFP	
R_{DONR}-GFP	GgggaccactttgtacaagaaagctgggtcGCTAGCTTTGTATAGTTCAT	eGFP nb 723-704		
F_{XhoI}-GUS	CCCTCGAGATGGTAGATCTGAGGGTAAATT	GUS nb 1-22, *Xho* I	pGD-GUS-3×Flag	
R_{ApaI}-GUS	GGGCCCTTGTTTGCCTCCCTGCTGCGGTTT	GUS nb 2024-2001, *Apa* I		
F_{SalI}-γb	GCGTCGACATGATGGCTACTTTCTCTTG	RNAγ nt 2081-2100, *Sal* I	pGDRm-γb	Subcellular localization
R_{ApaI}-γb	GGGGCCCCTTAGAAACGGAAGAAGAATC	RNAγ nt 2536-2516, *Apa* I		
F_{XhoI}-γb	CCCTCGAGATGATGGCTACTTTCTCTTG	RNAγ nt 2081-2100, *Xho* I	pGD-γb<br>pGD-$γb_{1-85}$<br>pGD-$γb_{1-127}$<br>pGD-$γb_{I130G}$<br>pGD-$γb_{1-85m}$	VSR activity assay
R_{BamHI}-γb	CGGGATCCTTACTTAGAAACGGAAGAAGA	RNAγ nt 2539-2519, *Bam*H I		
R_{BamHI}-$γb_{85}$	CGGGATCCTTAATGCTTTTGGCCACAGAACC	RNAγ nt 2335-2316, *Bam*H I		
R_{BamHI}-$γb_{127}$	CGGGATCCTTAAACCTCACTTTCAAGCTTAG	RNAγ nt 2461-2452, *Bam*H I		
F_{XbaI}-αa	GCTCTAGAAATGGCTAGCGATGAGATTGT	RNAα nt 92-111, *Xba* I	pSuper1300-αaGFP	Subcellular localization
R_{ApaI}-αa	GGGCCCCAACTATATGAGATTCAAAAG	RNAα nt 3505-3486, *Apa* I		

F_{XbaI}-γa	TGCTCTAGAAATGGATGTTGTGAAGAAATTCG	RNAγLIC nt 89-108, *Xba* I	pSuper1300-γaGFP	Subcellular localization
R_{KpnI}-γa	GGGGTACCCCACTCGAAGTCTTTTCTCAGC	RNAγLIC nt 2035-2014, *Kpn* I		
F_{KpnI}-γ	GGGGTACCAGATCTAACACCTCAAGTTGAATAT	RNAγLIC nt 1093-1117, *Kpn* I	pUC18-A_{RB}<br>pUC18-A_{RB}Invert<br>pUC18-A<br><br>pCaBS-$\gamma_{\Delta\gamma b}$<br>pCaBS-(+)γb_{PUM}<br>pCaBS-(-)γb_{PUM}	Northern blotting<br><br>RNA localization
R_{BamHI}-γ	CGGGATCCTGGTCTTCCCTTGGGGGACCGAAG	RNAγLIC nt 2811-2788, *Bam*H I		
F-T_{3809}	taattttgataGTTAAAAAAAAAAAAATGTTTGATC	RNAγLIC nt 2555-2582		
R-T_{3794}	aacttatctacaCTTAGAAACGGAAGAAGAATCATCA	RNAγLIC nt 2536-2512		
F_{Invert}-T_{3809}	aacttatctacaTAAAAAAAAAAAAATGTTTGATC	RNAγLIC nt 2557-2582		
R_{Invert}-T_{3794}	ttttgatagttCTTAGAAACGGAAGAAGAATCATCA	RNAγLIC nt 2536-2512		
F_{KpnI}-γb	GTGGTACCTTAACTACAAG	RNAγ nt 2109-2127, *Kpn* I	pCaBS-$\gamma_{\gamma b\Delta 86\text{-}127:GFP}$	Subcellular localization
R_{85}-γb	ATGCTTTTGGCCACAGAACCTAC	RNAγLIC nt 2334-2312		
R_{HpaI}-GFP	GAAGTTAACTTTGATTCCATTC	eGFP nb 494-473, *Hpa* I		
F_{LIC}-GFP	aaggaagttATGAGCAGTAAAGGAGAAGAACT	eGFP nb 1-23	pCaBS-$\gamma_{\Delta\gamma b:GFP}$	
R_{LIC}-GFP	aaccaccaccaccgtttaGCTAGCTTTGTATAGTT	eGFP nb 723-707		
$F_{\Delta\gamma bLIC}$-1	CATGCGAAGGTAAATACAGTAGATTTAAAC	RNAγLIC nt 2083-2054	pCaBS-$\gamma_{\gamma b86\text{-}127:GFP}$	
F_{86}-γb	GCGGATCTGTATGATTCACTTCTGAAACGTTC	RNAγLIC nt 2335-2366		

FΔγb	TGTATTTACCTTCGCACTGACTGGCTACTTTCTCTTGTGTG TGTTGTGG	RNAγLIC nt 2066-2114 ATGATG → ACTGACTG	pCaBS-$\gamma_{\gamma bATGm}$	Northern blotting
RΔγb	CAAGAGAAAGTAGCCAGTCAGTGCGAAGGTAAATACAG TAGATTTAAAC	RNAγLIC nt 2102-2054		
BS-3	GTATAGCTTGAGCATTACCGTC	RNAγ nt 1-22	pCaBS-$\gamma_{\gamma b1\text{-}127}$<br>pCaBS-$\gamma_{\gamma b:GFP}$	Northern blotting<br><br>Subcellular localization
R_{BamHI}-γ	CGGGATCCTGGTCTTCCCTTGGGGGACCGAAG	RNAγLIC nt 2811-2788, *Bam*H I		
F_{KpnI}-γb	GTGGTACCTTAACTACAAG	RNAγ nt 2109-2127, *Kpn* I	pCaBS-$\gamma_{\gamma bI130G}$	Northern blotting
R_{HpaI}-γb	AACCTCACTTTCAAGCTTAG	RNAγ nt 2461-2442, *Hpa* I		
F_{I130G}-γb	ACGCAGGACGTAAGTCCGTAGCTTCT	RNAγ nt 2462-2488		
R_{I130G}-γb	TACGTCCTGCGTTAACCTCACTTTCAA	RNAγ nt 2474-2448		
$F_{\Delta\gamma bLIC}$-2	CGTCCATGGGCGAAGGTAAATACAGTAGATTTAAAC	RNAγ nt 2079-2053	pCaBS-$\gamma_{\Delta\gamma b:P19}$	
$R_{\Delta\gamma bLIC}$-2	CGTACTAGTTAAGGAAGGGCCCGGTGGTGGTGGTTAA	RNAγ nt 2532-2559		
F_{NcoI}-P19	CATGCCATGGAACGAGCTATACAAGGAAACG	TBSV P19 nt 1-25, *Nco* I		
R_{ApaI}-P19	GCGGGCCCTTACTCGCTTTCTTTTTCGAAGGTTTG	TBSV P19 nt 519-492, *Apa* I		
F_{KpnI}-γ	GGGGTACCAGATCTAACACCTCAAGTTGAATAT	RNAγLIC nt 1093-1117, *Kpn* I		
F_{HpaI}-GFP	GCGTTAACATGAGCAGTAAAGGAGAAGA	eGFP nb 1-20, *Hpa* I	pCa-BS-$\gamma_{\gamma b1\text{-}127:GFP}$	Subcellular localization
R_{BamHI}-γ	CGGGATCCTGGTCTTCCCTTGGGGGACCGAAG	RNAγLIC nt 2811-2788, *Bam*H I		
F_{KpnI}-γ	GGGGTACCAGATCTAACACCTCAAGTTGAATAT	RNAγLIC nt 1093-1117, *Kpn* I		

F-GFP$_{39}$	ATGAGCAGTAAAGGAGAAGAACTTTTCACTGGAGTTGTC	eGFP nb 1-39	pCaBS-γ$_{γb1\text{-}85:GFP}$	Subcellular localization
R$_{86}$-γb$_{33}$	ATGCTTTTGGCCACAGAACCTACTCACGATTGG	RNAγLIC nt 2334-2302		
F$_{KpnI}$-γb	GTGGTACCTTAACTACAAG	RNAγ nt 2109-2127, *Kpn* I		
R$_{BamHI}$-γ	CGGGATCCTGGTCTTCCCTTGGGGGACCGAAG	RNAγLIC nt 2811-2788, *Bam*H I		
F$_{KpnI}$-γb$_{RE}$	CTTTCTCTTGTGTGTGTTGTGGTACCTTAACTACAAGTAC TTACTGTGGT	RNAγLIC nt 2090-2139, *Kpn* I		
F$_{GAD}$-γ	GTATTTGGTGGTGCTGATTCATTGATATTGTTTGATCAGG GATA	RNAγLIC nt 1577-1620	pCaBS-γ$_{γaGDDm}$	Northern blotting
R$_{GAD}$-γ	TATCAATGAATCAGCACCACCAAATACACAGAAATGTGC ATCT	RNAγLIC nt 1603-1561		
R$_{BamHI}$-γ	CGGGATCCTGGTCTTCCCTTGGGGGACCGAAG	RNAγLIC nt 2811-2788, *Bam*H I	pCa-BS-γ$_{γb1\text{-}85}$<br>pCa-BS-γ$_{γb1\text{-}85m}$	Northern blotting
R$_{ΔγbLIC}$-1	TAAGGAAGGGCCCGGTGGTGGTGGTTAA	RNAγLIC nt 2523-2560		
R$_{86}$-γb$_{33}$	ATGCTTTTGGCCACAGAACCTACTCACGATTGG	RNAγLIC nt 2334-2302		
F$_{BM26}$-γb	GATGTGAGCAAAACCATGTATATTCTGAAACAAG	RNAγLIC nt 2145-2177		
R$_{BM26}$-γb	TATACATGGTTTTGCTCACATCTCTTACCACAGTA	RNAγLIC nt 2165-2131		
F$_{MET}$-αa	acgtaccagattacgctcatATGGCTAGCGATGAGATTGTC	RNAα nt 92-112	pGADT7-αa$_{MET}$	Yeast two-hybrid
R$_{MET}$-αa	tatcgatgcccaccctctagaCAACTCAAATCTCAGAGGTCTA	RNAα nt 2593-2572		
F$_{HEL}$-αa	acgtaccagattacgctcatATGATTGACGGAGTTCCTGGCTG	RNAα nt 2594-2613	pGADT7-αa$_{HEL}$	Yeast two-hybrid
R$_{HEL}$-αa	tatcgatgcccaccctctagAAACTATATGAGATTCAAAAGTACGAA TAGG	RNAα nt 2593-2506		

F_{BamHI}-γb	CGGGATCCATGATGGCTACTTTCTCTTG	RNAγ nt 2081-2100, *Bam*H I	pGADT7-γb<br>pGBKT7-γb	Yeast two-hybrid
R_{EcoRI}-γb	CCATCGATTTACTTAGAAACGGAAGAAG	RNAγ nt 2538-2519, *Eco*R I		
$F_{\Delta seI}$-αa	GCATTAATATGGCTAGCGATGAGATTGT	RNAα nt 92-111, *Ase* I	pGADT7-αa<br>pGBKT7-αa	
R_{XbaI}-$αa_{AD}$	GCTCTAGATTAAACTATATGAGATTCAA	RNAα nt 3508-3489, *Xba* I		
F_{PstI}-$αa_{BD}$	AACTGCAGTTAAACTATATGAGATTCAA	RNAα nt 3508-3489, *Pst* I		
R_{EcoRI}-αa-1	CTCACCAAGTGGAATTCGCCC	RNAα nt 1711-1691, *Eco*R I		
F_{EcoRI}-αa	GGGCGAATTCCACTTGGTGAG	RNAα nt 1691-1711, *Eco*R I		
F_{NdeI}-$γb_{86-127}$	GGAATTCCATATGGCGGATCTGTATGATTCACT	RNAγLIC nt 2336-2355, *Nde* I	pGBKT7-$γb_{86-127}$	
R_{EcoRI}-$γb_{86-127}$	CGGAATTCAACCTCACTTTCAAGCTTAG	RNAγLIC nt 2461-2442, *Eco*R I		
F_{NdeI}-$γb_{128-152}$	GGAATTCCATATGAACGCAATACGTAAGTCCG	RNAγLIC nt 2462-2480, *Nde* I	pGBKT7-$γb_{128-152}$	
R_{EcoRI}-$γb_{128-152}$	CGGAATTCTTACTTAGAAACGGAAGAAGAATC	RNAγLIC nt 2539-2516, *Eco*R I		
F_{BamHI}-γb	CGGGATCCATGATGGCTACTTTCTCTTG	RNAγ nt 2081-2100, *Bam*H I	pSPYNE-γb<br>pSPYCE-γb	Bimolecular fluorescence complementation
R_{XhoI}-γb	CCCTCGAGCTTAGAAACGGAAGAAGAATC	RNAγ nt 2536-2516, *Xho* I		
F_{XbaI}-αa	GCTCTAGAATGGCTAGCGATGAGATTGT	RNAα nt 92-111, *Xba* I	pSPYNE-αa<br>pSPYCE-αa	
F_{SpeI}-αa	GGACTAGTAACTATATGAGATTCAAAAG	RNAα nt 3486-3505, *Spe* I		

F_{SpeI}-γa	CGGACTAGTATGGATGTTGTGAAGAAATT	RNAγLIC nt 89-108, *Spe* I	pSPYNE-γa<br>pSPYCE-γa	Bimolecular fluorescence complementation
R_{SalI}-γa	GCGTCGACCCACTCGAAGTCTTTTCTCA	RNAγLIC nt 2035-2016, *Sal* I		
F-$αa_{LRSV}$	gctcaggcctggcgcgccactagtATGGCGAGTGATGAGATCGT	LRSV RNAα nt 88-107	pSPYNE-$αa_{LRSV}$<br>pSPYCE-$αa_{LRSV}$	
R-$αa_{LRSV}$	catcccgggagcggtaccctcgagATTAACAATATGTTCTCTGAAAATA CTTACAGG	LRSV RNAα nt 3510-3478		
F-$αa_{PSLV}$	gctcaggcctggcgcgccactagtATGGCAAGTGACGAGATTGT	PSLV RNAα nt 116-135	pSPYNE-$αa_{PSLV}$<br>pSPYCE-$αa_{PSLV}$	
R-$αa_{PSLV}$	catcccgggagcggtaccctcgagCTCTACAATATGCGATTGGAAAGTC	PSLV RNAα nt 3574-3550		
F-$γb_{LRSV}$	gctcaggcctggcgcgccactagtATGGCATCTTCACCTAATGT	LRSV RNAα nt 1979-1988	pSPYNE-$γb_{LRSV}$<br>pSPYCE-$γb_{LRSV}$	
R-$γb_{LRSV}$	catcccgggagcggtaccctcgagAAGCTTAGAACTATTGCGGAGA	LRSV RNAα nt 2410-2389		
F-$γb_{PSLV}$	gctcaggcctggcgcgccactagtATGTCAACCGACTTGTGTTC	PSLV RNAα nt 2345-2364	pSPYNE-$γb_{PSLV}$<br>pSPYCE-$γb_{PSLV}$	
R-$γb_{PSLV}$	catcccgggagcggtaccctcgagGAGTTTACTTAGTTTGAAAAAATC CGCC	PSLV RNAα nt 2875-2848		
F_{BamHI}-γb	CGGGATCCATGATGGCTACTTTCTCTTG	RNAγ nt 2081-2100, *Bam*H I	pGEX2T-γb<br>pGEX2T-$γb_{BM26}$	Protein expression
R_{EcoRI}-γb	CCATCGATTTACTTAGAAACGGAAGAAG	RNAγ nt 2538-2519, *Eco*R I		
F_{NdeI}-αa	GGAATTCCATATGGTGGCCGTTCCATTGAACGC	RNAα nt 2252-2271, *Nde* I	pDB.His.MBP-$αa_{HEL}$	
R_{XhoI}-αa	CCGCTCGAGAACTATATGAGATTCAAAAGTACG	RNAα nt 3505-3482, *Xho* I		
$F_{HindIII}$-BS(-)	CCAAGCTTTGGTCTTCCCTTGGGGGACC	RNAγLIC nt 2790-2070	pSPT19-BS(-)	Probe preparation
R_{EcoRI}-BS(-)	CGGGATCCTGTTTGATCAGATCATTCAA	RNAγLIC nt 2551-2571		

$F_{HindIII}$-αa	CCCAAGCTTGCTTGGTGCCTCAGAAATTCTCGC	RNAα nt 2872-2895, *Hin*d III	pSPT19-αaA<br>pSPT18-αaA	Probe preparation
R_{EcoRI}-αa-2	CGGAATTCTTAAACTATATGAGATTCAAAAGTACG	RNAα nt 3508-3482, *Eco*R I		
F-P19	ATGGAACGAGCTATACAAGGAAAC	TBSV P19 nt 1-24		PCR analysis of p19 transgenic plant
R-P19	CTCGCTTTCTTTTTCGAAGGTTTGAG	TBSV P19 nt 515-489		
BS-10	GGTGCTTGATGCTTTGGATAAGG	RNAγ nt 1862-1884		Screening of positive clones
BS-32	TGGTCTTCCCTTGGGGGAC	RNAα, β, γ 3′-terminus		

[a] Underlined letters indicate restriction enzyme sites, lowercase letters indicate sequence used for recombination. Shadowed letters indicate extra nucleotides introduced to the original sequence

[b] Numbers correspond to target nucleotide positions, a reverse order of numbers indicates that the primer is complementary to the target sequences. RNAα, β, and γ indicate the BSMV components unless specifically noted.
