# Supplementary material for: The Barley stripe mosaic virus γb protein promotes chloroplast-targeted replication by enhancing unwinding of RNA duplexes
Source: PLoS Pathog. 2017 Apr 7;13(4):e1006319. doi: 10.1371/journal.ppat.1006319 (PMC5397070; doi:10.1371/journal.ppat.1006319)
Supplement: S2 Table — Underlined letters constitute the complementary regions of the partial dsRNA duplex. (PDF) [file ppat.1006319.s002.pdf]

S2 Table. Sequences of single-stranded RNA used for preparation of the partial dsRNA duplex.

	**Sequence (5'-3')**
**Template RNA strand (149 nt)**	5′-GAGACCCAAGCUUGCAUGCCUGCAGGUCGACUCUAGAGGAUCCCCGGGUACCGAGCUCGAAUUCCG<br>UGUAUUCUAUAGUGUCACCUAAAUCGUAUGUGUAUGAUACAUAAGGUUAUGUAUUAAUUGUAGCCGC<br>GUUCUAACGACAAUAU-3′
**Release RNA strand (55 nt)**	5′-CGAGCUCGGUACCCGGGGAUCCUCUAGAGUCGACCUGCAGGCAUGCAAGCUUGGG-3′
