# Supplementary figures and images for: The Barley stripe mosaic virus γb protein promotes chloroplast-targeted replication by enhancing unwinding of RNA duplexes
Source: PLoS Pathog. 2017 Apr 7;13(4):e1006319. doi: 10.1371/journal.ppat.1006319 (PMC5397070; doi:10.1371/journal.ppat.1006319)

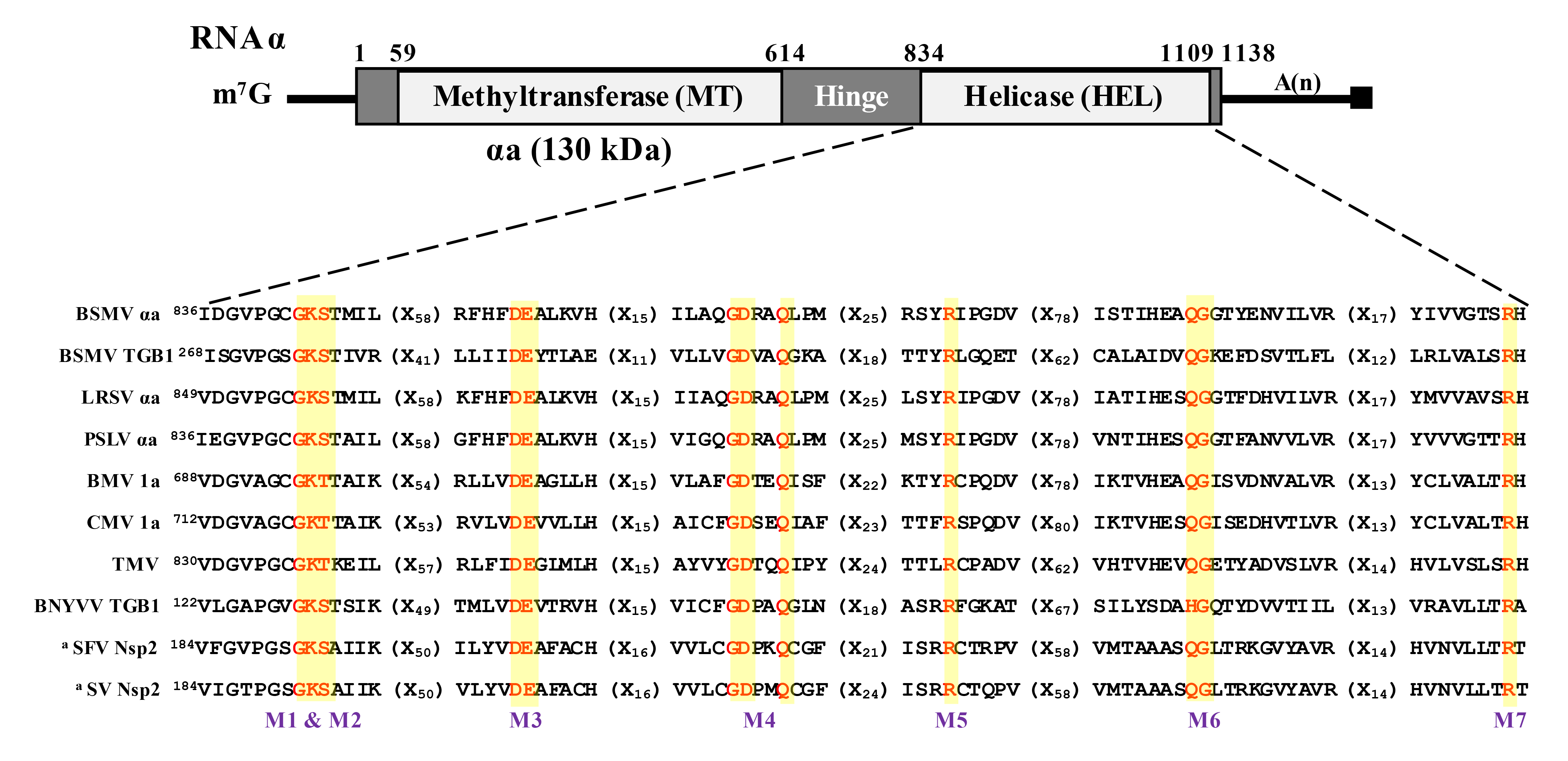

Supplement: S1 Fig — Common or related amino acid residues are shown in red text and highlighted in yellow. “Xn” indicates the number of variant amino acids between the conserved domains. (TIF) [file ppat.1006319.s003.tif]

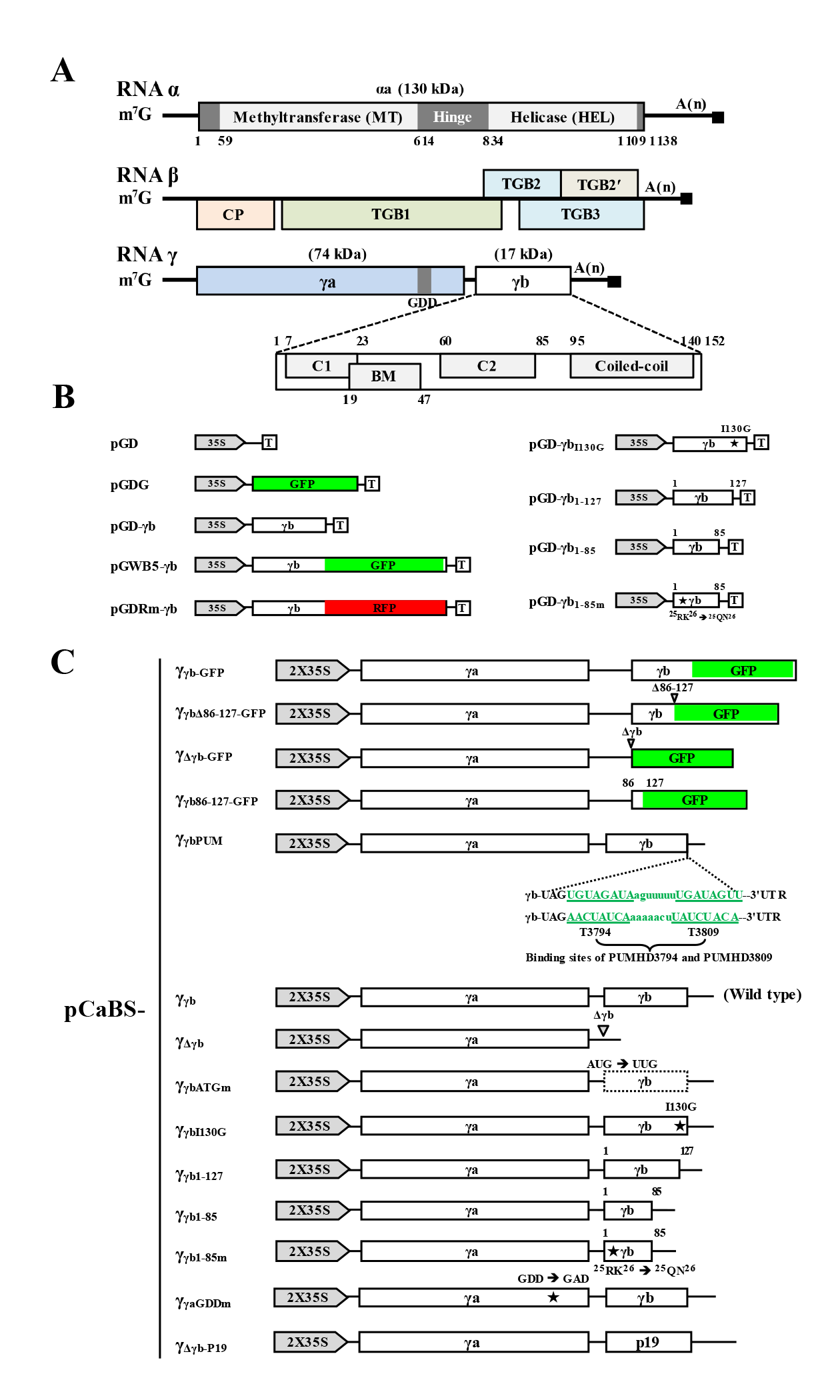

Supplement: S2 Fig — Panel A: BSMV Genome organization with functional regions of the γb protein highlighted. Panel B: Illustration of γb plasmids used for transient expression of γb and various γb derivatives in N. benthamiana leaf tissues. Panel C: Diagram of various pCaBS plasmids harbored in A. tumefaciens for delivery of infectious BSMV RNA derivatives after agroinfiltration of leaf tissues. RNAs transcribed in cells after agroinfiltration of the pCaBS-α, pCaBS-β, and pCaBS-γ or pCaBS-γΔγb-GFP vectors were sometimes designated BSMV RNAs α, β, γ, γΔγb-GFP, etc. Note: The γa replicase was inactivated by mutating the GDD motif to GAD. (TIF) [file ppat.1006319.s004.tif]

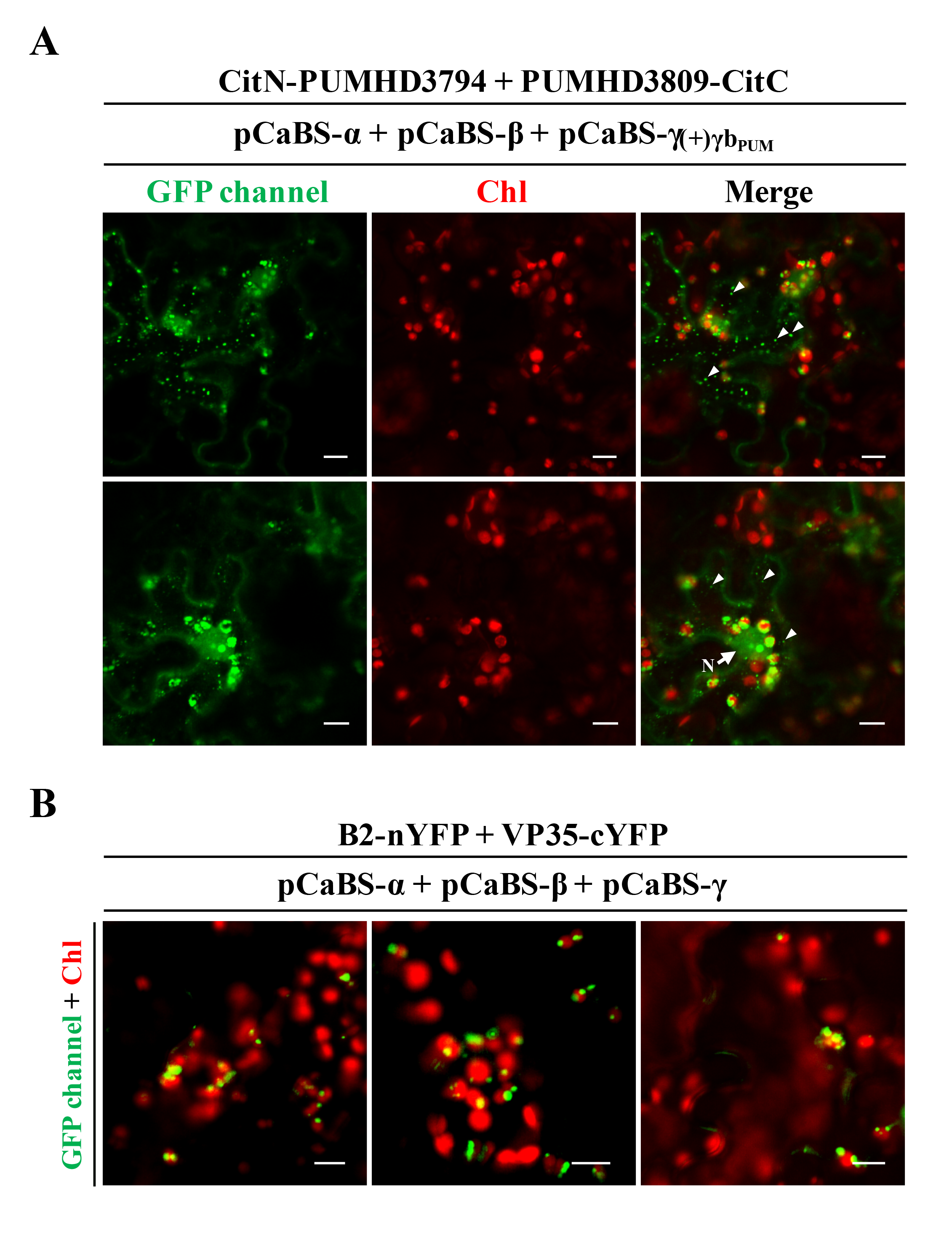

Supplement: S3 Fig — Panel A: Leaves were agroinfiltrated with RNAα + RNAβ + RNAγ(+)γbPUM as in Fig 1. The white arrow indicates the nucleus (N). Arrowheads indicate the cytoplasm-localized plus-strand BSMV RNAs. Scale bar, 10 μm. Panel B: Symptomatic leaves of BSMV-infected N. benthamiana plants were co-infiltrated with the split YFP-tagged FHV B2 and Marburg virus VP35 proteins. Images are the overlay of GFP channel and chlorophyll autofluorescence (Chl). Scale bar, 10 μm. Note: The figure organization and relevant figure designations can be found in the Fig 1 legend. (TIF) [file ppat.1006319.s005.tif]

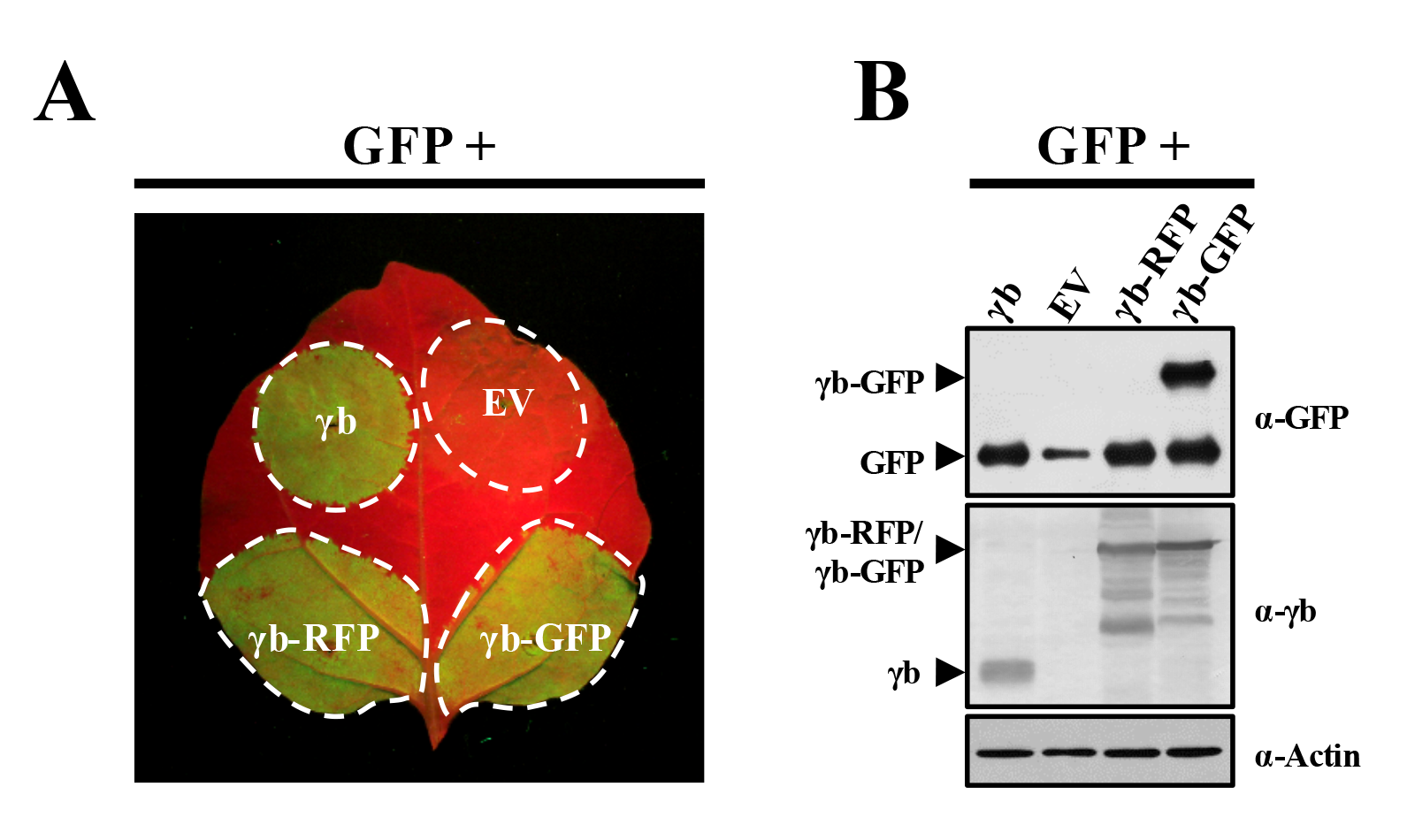

Supplement: S4 Fig — Panel A: N. benthamiana leaves were agroinfiltrated with A. tumefaciens harboring plasmids (see S2B Fig) for expression of γb (positive control), γb-GFP or γb-RFP for the spot silencing assay, and then photographed under long wavelength UV illumination at 3 dpi. (EV = empty pGD vector for use as a negative control). Panel B: Western blot analysis of the expressed proteins in agroinfiltrated leaves. Bands corresponding to the reporter proteins were shown on the left (arrowheads), and antibodies used for protein detection are shown on the right side of each blot. Equal protein loading is suggested by the similar amounts of actin protein (bottom panel). (TIF) [file ppat.1006319.s006.tif]

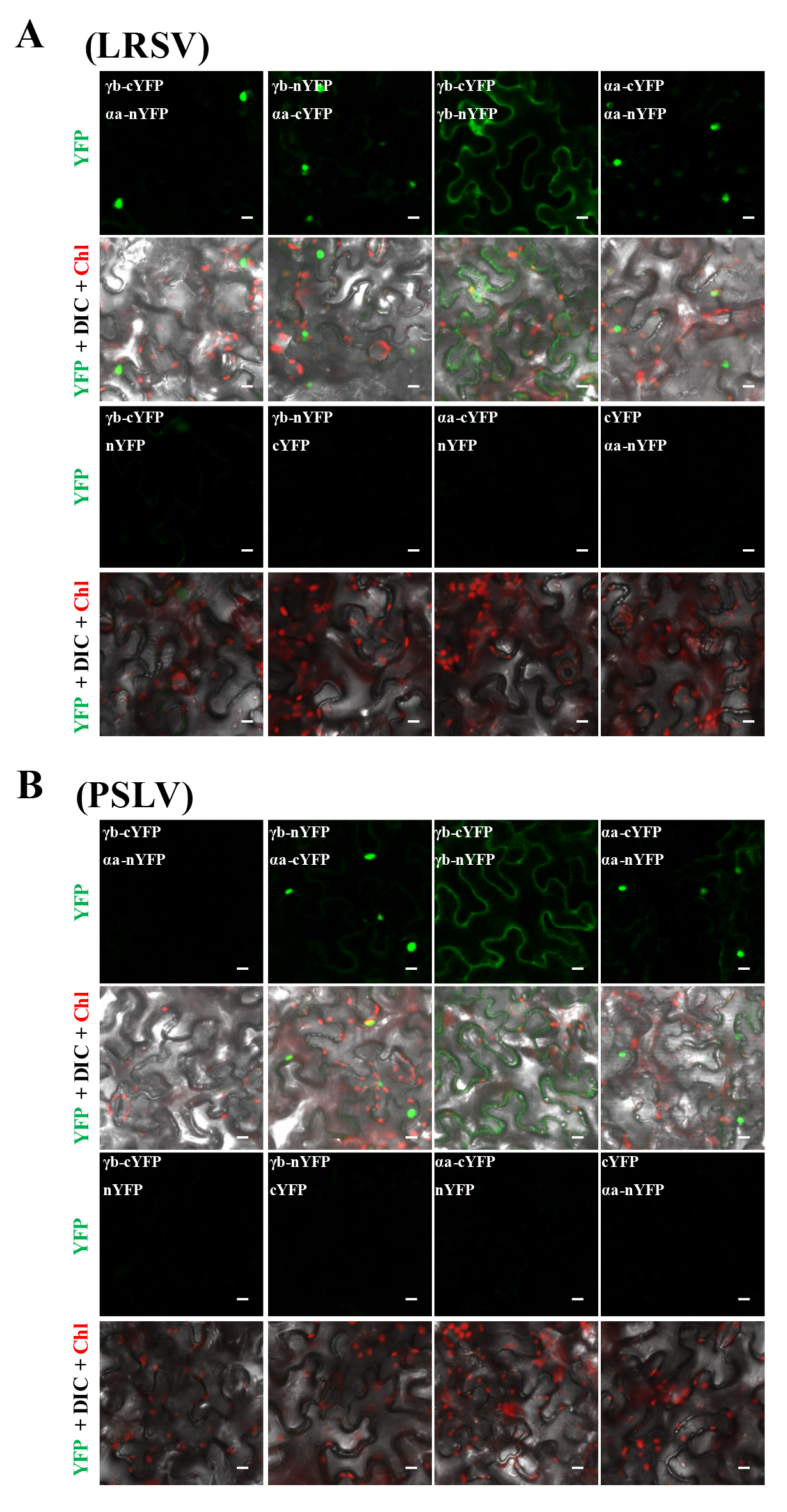

Supplement: S5 Fig — Panels A and B: BiFC assays to detect interactions between the αa and γb proteins of LRSV and PSLV. The experimental design was described in the legend to Fig 3A, and confocal microscopic analyses were carried out at 3 dpi. Various constructs used for agroinfiltration are indicated on top left corner of each panel, while the channels used for imaging are shown on the left. The YFP signal is false-colored green. DIC, Differential interference contrast. Chl, chlorophyll autofluorescence (in red). Scale bar, 10 μm. Note: The LRSV and PSLV αa and γb binding and self-interactions are similar to those of BSMV. However, the fluorescence locations of both the LRSV and PSLV positive αa-γb interactions appear to be distinct from chloroplast autofluorescence, suggesting that these viruses and BSMV may replicate in different subcellular sites. (TIF) [file ppat.1006319.s007.tif]

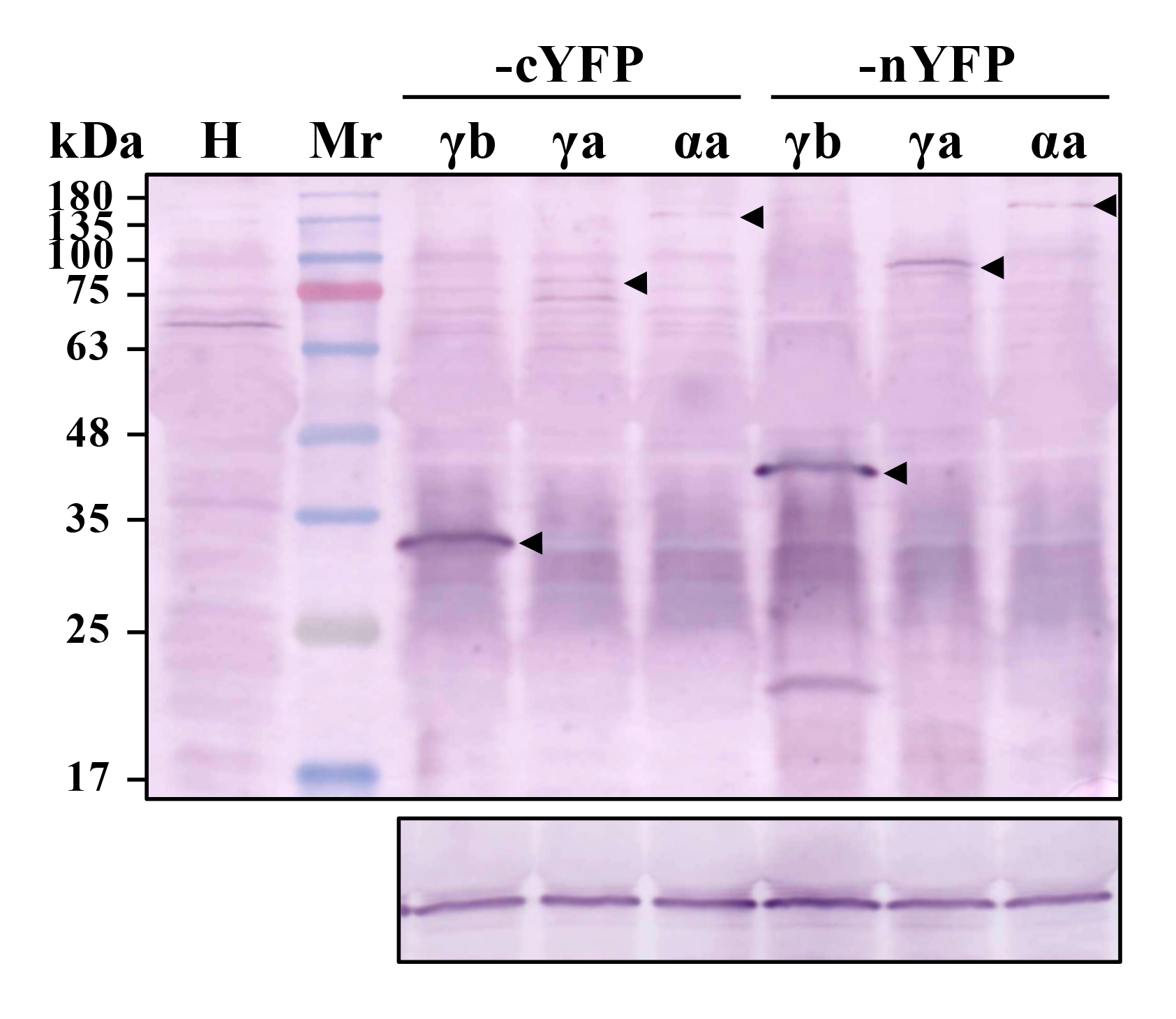

Supplement: S6 Fig — Detection of half-YFP fusion proteins in agroinfiltrated leaves at 3 dpi. Various combinations of the -cYFP and -nYFP derivatives shown at the top of the panel correspond to those shown in Fig 3A. Sizes (in kDa) of molecular weight markers (Mr) are shown on the left and the antibodies used for detection are shown on the right. Arrowheads indicate the target proteins. H, Healthy leaf control. Equal protein loading was assessed by Actin detection (bottom panel). (TIF) [file ppat.1006319.s008.tif]

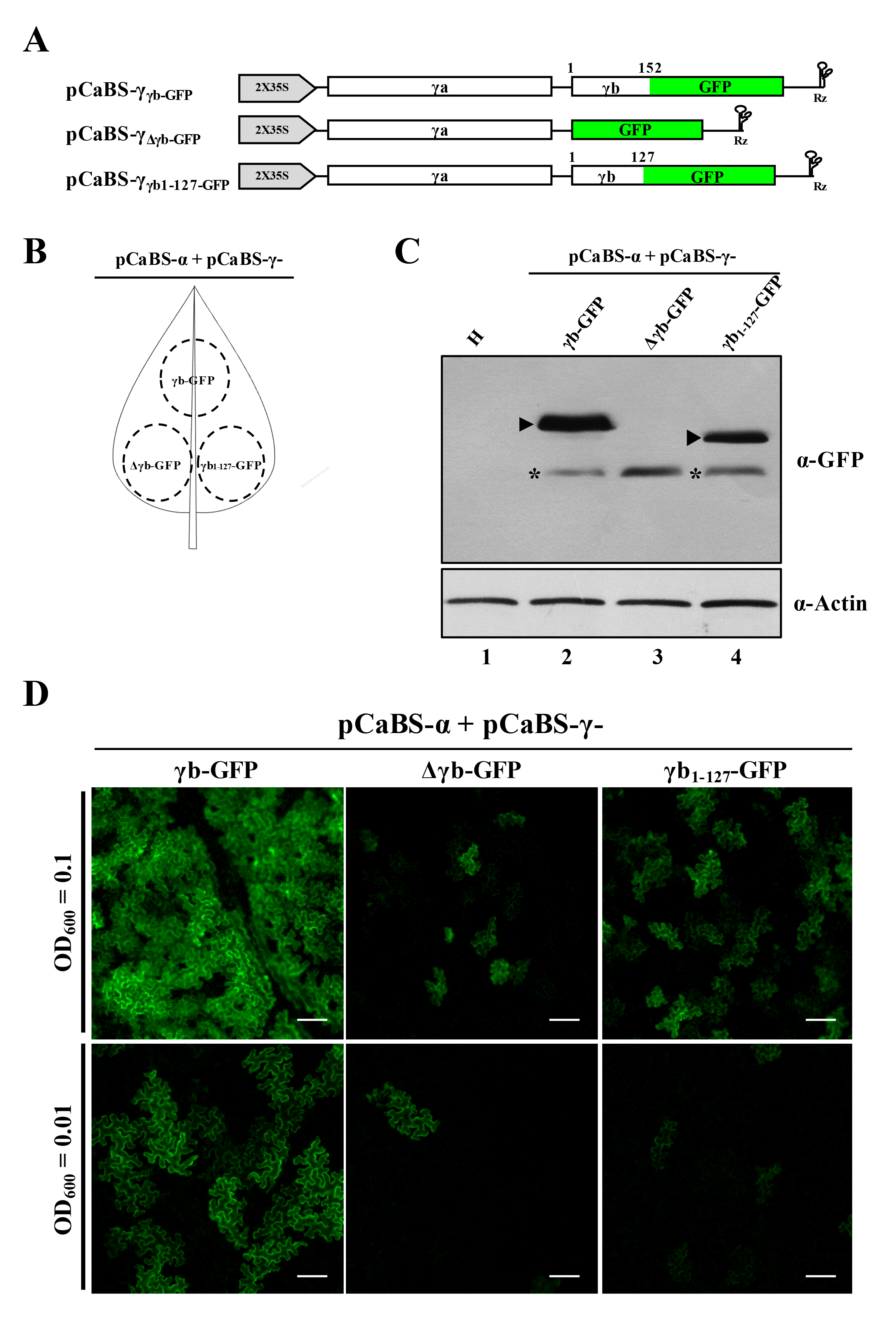

Supplement: S7 Fig — Panel A: Schematic representation of RNAγ GFP constructs. Panel B: Diagram of the leaves co-infiltrated with RNAα and the RNAγ GFP variants illustrated in S7A Fig. Panel C: Western blot analysis of total protein samples from leaf tissues co-infiltrated with RNAα and RNAγ-derived constructs (OD600 = 0.1) using antibodies against GFP. Equal protein loading was assessed by Actin detection (bottom panel). H, healthy leaf control. Note: Small bands (asterisks) appearing below the γb-GFP fusion variants (arrowheads) in lanes 2 and 4 probably were due to free GFP expression mediated by recombination of RNAγγbGFP variants. Equal protein loading was assessed by Actin detection (bottom panel). Panel D: Confocal microscopic analysis of leaf tissues co-infiltrated with RNAα and RNAγ-derived constructs. The concentrations of Agrobacterium cells used for agroinfiltration are shown on the left sides of the horizontal images. Scale bar, 100 μm. (TIF) [file ppat.1006319.s009.tif]

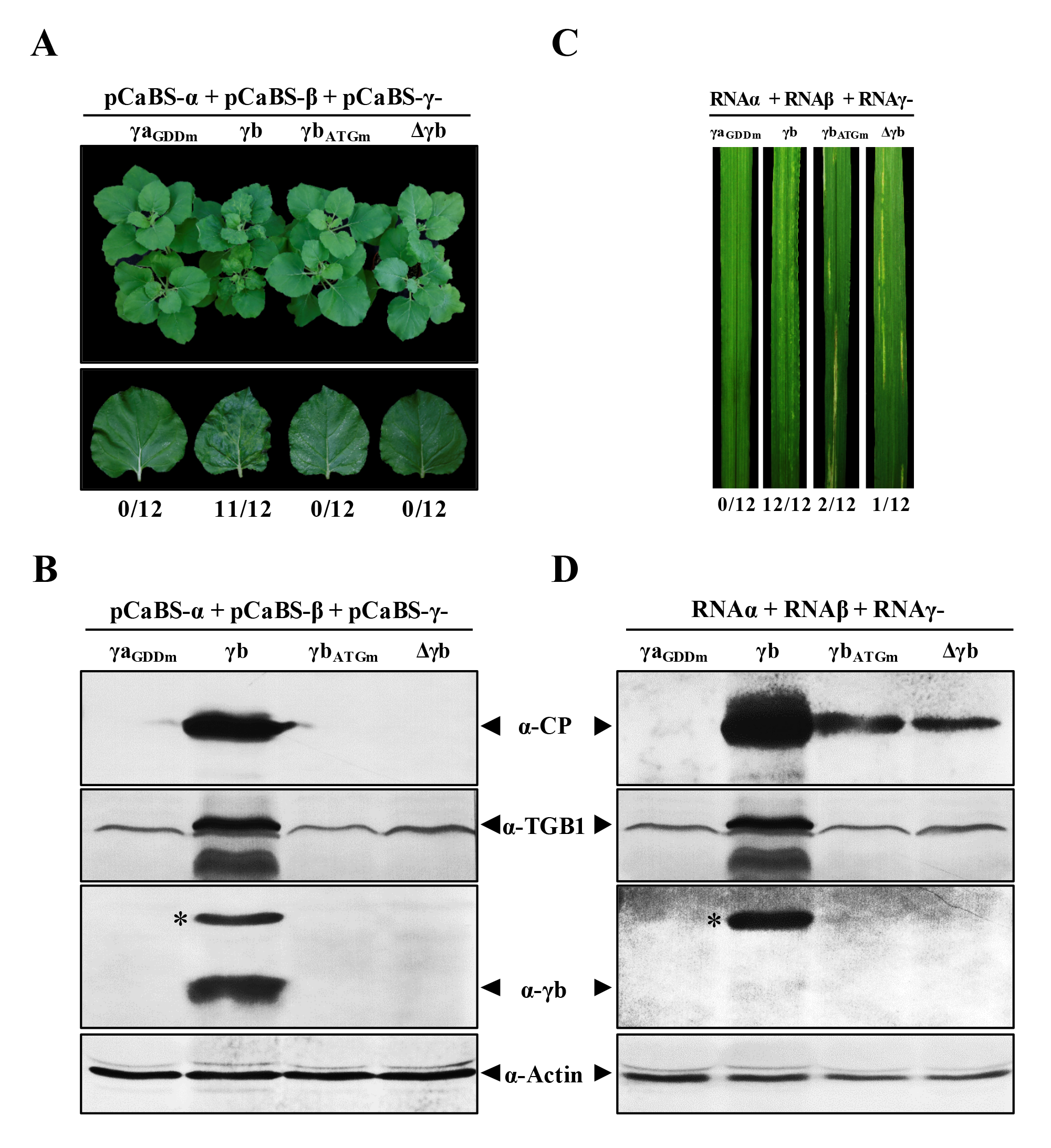

Supplement: S8 Fig — Panel A: Symptom phenotypes of N. benthamiana plants co-infiltrated with Agrobacteria expressing RNAα, RNAβ, and various RNAγ-derivatives shown above the upper image. The lower image shows representative symptoms of upper uninoculated leaves corresponding to the plants shown above. The numbers beneath the lower panel indicate the numbers of plants expressing systemic symptoms among 12 inoculated N. benthamiana plants at 24 dpi. Note: Only those plants inoculated with wtBSMV exhibit visible symptoms. Panel B: Western blot analysis of protein samples from upper uninoculated leaves shown in the lower panel of S8A Fig. Antibodies used for detection are indicated on the right. Equal protein loading was assessed by Actin detection (bottom panel). Panel C: Systemic symptoms of barley leaves mechanically co-inoculated with in vitro transcripts of RNAα, RNAβ, and various RNAγ-derivatives shown above each image. The numbers beneath the lower images indicate the numbers of barley plants that display systemic symptoms among 12 inoculated plants at 54 dpi. Panel D: Western blot analysis of the total protein samples from upper uninoculated leaves of the barley leaves as shown in S8C Fig. Antibodies used for detection are indicted on the left. Equal protein loading was assessed by Actin detection (bottom panel). Arrowheads between the two horizontal panels indicate the target band of corresponding viral protein. Note: a band that we suspect to be a γb doublet (asterisk) is present in panels B and D. In addition, the γb mutant derivatives result in very low levels of expression of the CP and TGB1 as has frequently been observed previously. (TIF) [file ppat.1006319.s010.tif]

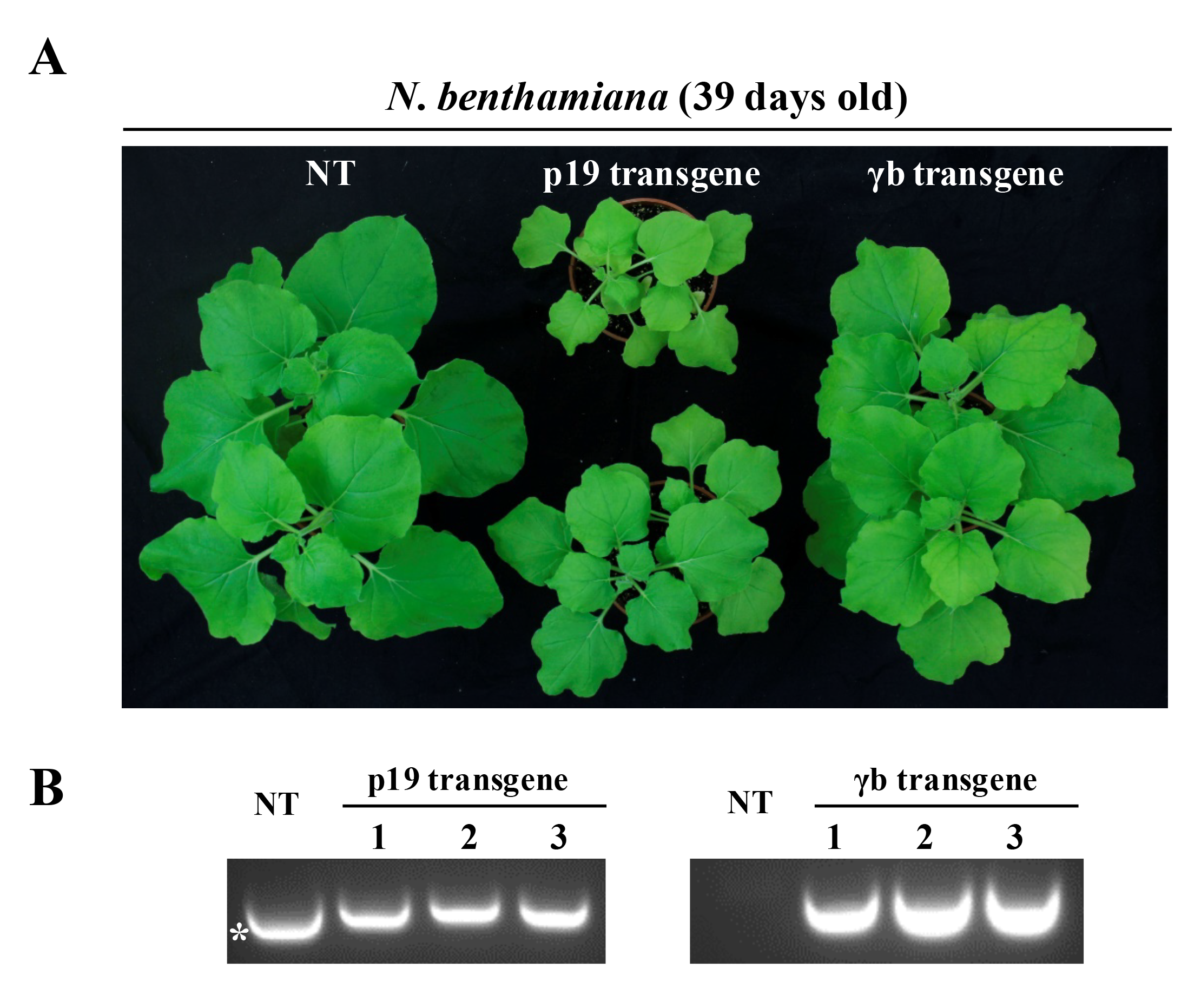

Supplement: S9 Fig — Phenotypic observations (Panel A) and PCR amplification (Panel B) of p19- and γb-transgenes from N. benthamiana plants. NT, non-transgenic. The asterisk indicates the non-specific band. (TIF) [file ppat.1006319.s011.tif]

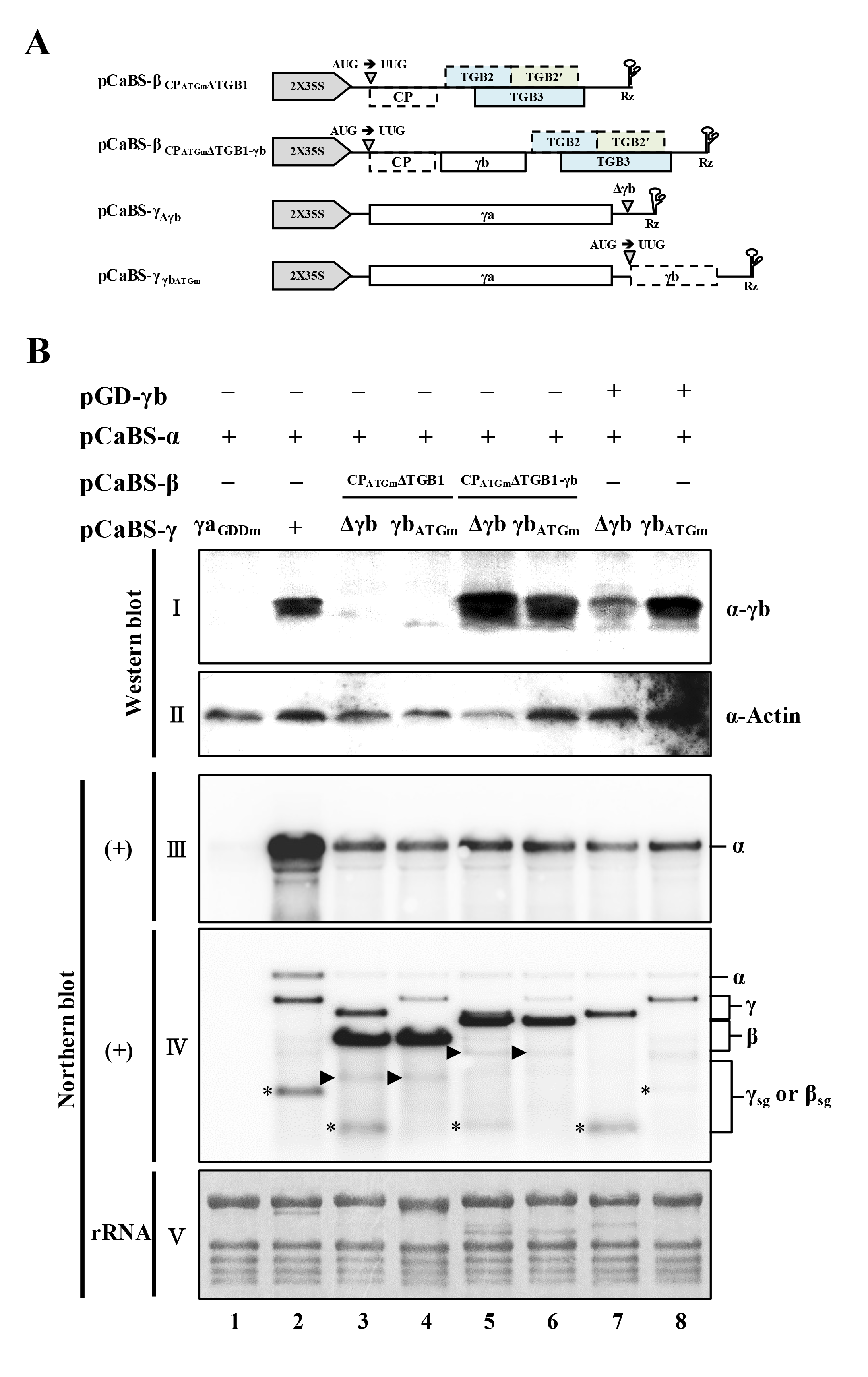

Supplement: S10 Fig — Panel A: Schematic representation of plasmids used for agroinfiltration of N. benthamiana. Panel B: Molecular analysis of the replication of γb-deficient BSMV in the agroinfiltrated N. benthamiana leaves by Western blot (I and II), and Northern blot (III-V). Lanes 1–2, negative and positive control leaves agroinfiltrated with Agrobacteria strains for co-expression of wild-type RNAα + RNAγγaGDDm, and wild-type RNAα + wild-type RNAγ. Lane 3, leaves co-expressing RNAα, γb-deficient RNAγ (Δγb) or γbATGm), and a modified RNAβ (CPATGmΔTGB1) mutant unable to express TGB1, TGB2, and CP. Lane 4, co-expressions identical to Lane 3 except that γbATGm was included in the infiltration mixture instead of the Δγb mutant. Lane 5, leaves co-infiltrated for expression of RNAα, a γb-deficient mutant RNAγ (Δγb) and a modified RNAβ with γb substituted for the TGB1 ORF (CPATGmΔTGB1-γb shown in S10A Fig). Lane 6, identical to lane 5 except that the infiltration mixture included γbATGm instead of Δγb. Lanes 7 and 8, leaves co-infiltrated for expression of RNAα, and γb and the γb-deficient RNAγ mutants Δγb or γbATGm, respectively. For Western blot analysis, the γb- and Actin-specific antibodies for protein identification are shown on the right. Equal protein loading was evaluated by Actin detection (panel II). For Northern blot analysis, the probes used for detection of the plus-strand (+) RNAs are shown on the left. Bands corresponding to RNAα, RNAβ, RNAγ, and subgenomic RNAγ (see *-labeled bands, γsg), or subgenomic RNAβ (see arrowhead-labeled bands, βsg) are indicated along the right side of each panel. Methylene blue staining of rRNAs was used as a loading control (panel V). The experiments were independently repeated twice and similar results were obtained. (TIF) [file ppat.1006319.s012.tif]

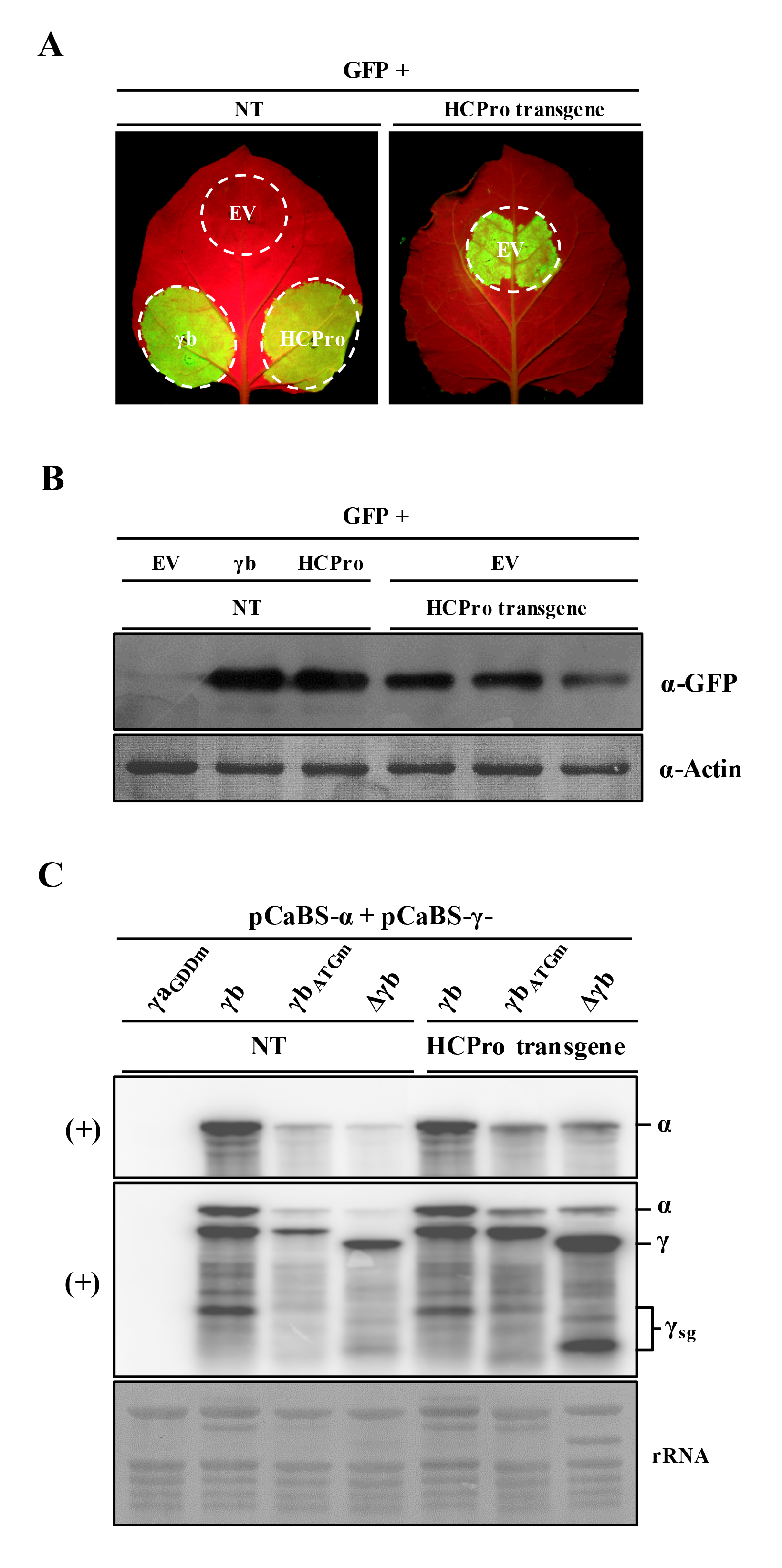

Supplement: S11 Fig — Panel A: Gene silencing suppression abilities of HCPro-transgenic N. benthamiana. Non-transgenic (NT) and HCPro-transgenic plants were co-infiltrated with 35S-GFP plus the empty pGD vector (EV) and observed at 3 dpi under UV illumination. Transient coexpression of 35S-GFP and HCPro, and 35S-GFP and γb in non-transgenic plants served as positive controls. Panel B: Western blot analysis of total protein samples from agroinfiltrated N. benthamiana leaves using a GFP-specific antibody. Equal protein loading was assessed by Actin detection. Panel C: Northern blot analysis of plus-strand BSMV RNAα (Top blot) or BSMV α, and γ plus-strand RNAs (Bottom blot). N. benthamiana leaves were agroinfiltrated with Agrobacteria strains for expression of RNAα and the RNAγ-derivatives shown above the panel lanes. Bands indicative of RNAα, RNAγ, and sgRNAγ (γsg) are indicated on the right. Methylene blue staining of rRNAs was used as a loading control. Blots are representative of two independent experiments with similar results. (TIF) [file ppat.1006319.s013.tif]
